# Cardiolipotoxicity, Inflammation, and Arrhythmias: Role for Interleukin-6 Molecular Mechanisms

**DOI:** 10.3389/fphys.2018.01866

**Published:** 2019-01-07

**Authors:** Alessandra Alí, Mohamed Boutjdir, Ademuyiwa S. Aromolaran

**Affiliations:** ^1^Cardiovascular Research Program, VA New York Harbor Healthcare System, Brooklyn, NY, United States; ^2^Department of Medicine, State University of New York Downstate Medical Center, Brooklyn, NY, United States; ^3^Department of Cell Biology, State University of New York Downstate Medical Center, Brooklyn, NY, United States; ^4^Department of Pharmacology, State University of New York Downstate Medical Center, Brooklyn, NY, United States; ^5^Department of Molecular and Developmental Medicine, University of Siena, Siena, Italy; ^6^Department of Medicine, New York University School of Medicine, New York, NY, United States

**Keywords:** interleukin-6, cytokines, ion channel, arrhythmias, inflammation, saturated free fatty-acids, toll-like receptors, lipotoxicity

## Abstract

Fatty acid infiltration of the myocardium, acquired in metabolic disorders (obesity, type-2 diabetes, insulin resistance, and hyperglycemia) is critically associated with the development of lipotoxic cardiomyopathy. According to a recent Presidential Advisory from the American Heart Association published in 2017, the current average dietary intake of saturated free-fatty acid (SFFA) in the US is 11–12%, which is significantly above the recommended <10%. Increased levels of circulating SFFAs (or lipotoxicity) may represent an unappreciated link that underlies increased vulnerability to cardiac dysfunction. Thus, an important objective is to identify novel targets that will inform pharmacological and genetic interventions for cardiomyopathies acquired through excessive consumption of diets rich in SFFAs. However, the molecular mechanisms involved are poorly understood. The increasing epidemic of metabolic disorders strongly implies an undeniable and critical need to further investigate SFFA mechanisms. A rapidly emerging and promising target for modulation by lipotoxicity is cytokine secretion and activation of pro-inflammatory signaling pathways. This objective can be advanced through fundamental mechanisms of cardiac electrical remodeling. In this review, we discuss cardiac ion channel modulation by SFFAs. We further highlight the contribution of downstream signaling pathways involving toll-like receptors and pathological increases in pro-inflammatory cytokines. Our expectation is that if we understand pathological remodeling of major cardiac ion channels from a perspective of lipotoxicity and inflammation, we may be able to develop safer and more effective therapies that will be beneficial to patients.

## Introduction

The heart utilizes free fatty acids (FFAs) to generate a significant proportion of its energy source. Under normal conditions in the heart, circulating lipid is maintained through a regulated balance between cardiac lipid uptake and oxidation. During pathological situations, such as in metabolic disorders, fat levels exceed the storage capacity of adipocytes. The excessive circulating FFA levels underlie fatty acid infiltration of cardiomyocytes (van Herpen and Schrauwen-Hinderling, 2008). Therefore, in metabolic disorders including obesity, type 2 diabetes (T2D) and insulin resistance, FFA levels increase to >1 mM from normal levels (0.2–0.8 mM) (Altarejos et al., 2005). The excess amounts of FFAs are subsequently converted into triglycerides and stored as lipid droplets. The chronic accumulation of lipid droplets and/or lipid metabolic intermediates within the myocardium may directly disrupt cardiac function associated with lipotoxic cardiomyopathy (Szczepaniak et al., 2007). The expectation is that over time the constant and continuous metabolic stress is likely to lead to heart failure. Thus, metabolic-related studies that focus on understanding the progressive deterioration of myocardial structural and electrical integrity are needed. Investigations of molecular mechanisms that occur in the initial phase of the disease state are especially important.

In recent years, there have been studies that have supported this notion. For example, an elegant study conducted by Taegtmeyer and others (Sharma et al., 2004), used both failing human hearts from diabetes and obesity patients, and hearts from ZDF rats with intramyocardial lipid deposition. It was demonstrated that myocardial lipid accumulation led to an upregulation of pathological markers of impaired fatty acid metabolism (peroxisome proliferator-activated receptor alpha, PPARα), contractility (myosin heavy chain beta, MHC-β), and inflammatory response (tumor necrosis factor alpha, TNF-α). Genetically manipulated mice, with altered cardiac-specific metabolic pathways (lipid transport and storage) display decreased mitochondrial biogenesis (Glenn et al., 2011) and impaired diastolic function (Chiu et al., 2005; Flagg et al., 2009). We have also shown that high-fat diet induced obesity caused atrial electrical remodeling associated with vulnerability to atrial fibrillation (AF) (Aromolaran et al., 2016).

Furthermore, fatty acid metabolism has been investigated as a contributing factor in the pathogenesis of lipotoxic cardiomyopathy and heart failure (Tsushima et al., 2018). These studies assessed the impact of altered expression of genes involved in regulation of FFA uptake and metabolism (acyl-CoA synthetase), FFA transport (FATP1) (Chiu et al., 2005), and lipid utilization (adipose triglyceride lipase, ATGL) (Hirano et al., 2008; Hoy et al., 2011). For example, Goldberg’s group previously showed that mice fed a normal diet but overexpressing cardiac-specific lipoprotein lipase (LpL), an enzyme that hydrolyzes circulating triglycerides and releases FFAs, displayed dilated hearts with left ventricular systolic dysfunction (Yagyu et al., 2003). Schaffer and others have also reported that mice overexpressing cardiac FATP1 developed cardiac phenotypes like those seen in T2D and obese animals (Chiu et al., 2005). Collectively, these studies provide convincing evidence that cardiomyocyte-specific lipid deposition is critically associated with cardiac abnormalities in metabolic disorders.

Despite the clinical implications of lipid accumulation in the heart (Poirier et al., 2006; He et al., 2017; Anstee et al., 2018; De Coster et al., 2018), the underlying molecular mechanisms are poorly understood. A major limitation could be due to the complexity associated with the involvement of multiple signaling pathways which include: (1) direct modulation of ion channel function by FFAs, and (2) FFA activation of the toll-like receptor (TLR) and nuclear factor kappa-light-chain-enhancer of activated B cells (NFκB) leading to secretion of pro-inflammatory cytokines (Huh et al., 2016) and subsequent cardiac electrical remodeling. Previous reports have demonstrated that FFAs increase inflammation (Lundman et al., 2007), while genetic knockdown of TLR is protective in mice fed a high-fat diet rich in palmitate (Davis et al., 2008). Despite the clinical implications of these findings, there is a paucity of studies that address modulation of ion channels through activation of the SFFAs/TLR/NFκB/cytokine pathway in heart.

Here we review the recent developments regarding myocardial lipid accumulation as a mechanism contributing to cardiac ion channel dysfunction. We further discuss a role for pro-inflammatory cytokines, and more importantly interleukin-6 (IL-6), a dynamic and multifunctional cytokine with well-defined pro-inflammatory and anti-inflammatory functional characteristics (Szabo-Fresnais et al., 2010; Mihara et al., 2012; Lazzerini et al., 2017a; Tanaka et al., 2017). The knowledge that the immune system is an important component of dysregulated metabolic pathways is a key step to understanding the pathogenesis of heart failure in patients. We provide our viewpoint on whether targeting cytokine signaling pathways in the heart may be a different mechanism to treat arrhythmias. The possibility of this mechanism-based approach is strengthened by a recent report by Tyler’s group (Lewis et al., 2018). The investigators describe the feasibility of using non-invasive hyperpolarized magnetic resonance imaging with [1-^13^C]pyruvate as a marker of cytokine production. This non-invasive test will make it possible to screen obese and diabetic patients for early signs of heart failure.

## Molecular Signaling Pathways of Cardiolipotoxicity That Promote Cardiac Inflammation

### Free-Fatty Acids

There is increasing evidence that dietary FFAs are a critical and independent predictor of metabolic disorders including insulin resistance, T2D and obesity (Kien et al., 2005; Park and Goldberg, 2012), and related cardiac dysfunction (Haim et al., 2010; Shao et al., 2013; O’Connell et al., 2015; Aromolaran et al., 2016; Anumonwo and Herron, 2018). This association underscores the importance of studies that provide vigorous and comprehensive molecular insights into the structural determinants of the functional properties of FFA as well as FFA-activated signaling pathways in heart. FFAs are characterized by a straight chain of carbon atoms with both a carboxylic (COOH) and a methyl (CH_3_ or omega, ω) end (Rennison and Van Wagoner, 2009) and are generally classified based upon the level of saturation on the carbon atoms. Accordingly, FFAs are classified into three main groups: (1) saturated fatty acids (SFAs) that do not contain double bonds (C16:0 and C18:0), (2) monounsaturated fatty acids (MUFAs), that contain only one double bond (C18:1), and (3) polyunsaturated fatty acids (PUFAs), that contain at least two double bonds (Huang et al., 2018). PUFAs, are divide into two classes namely: ω-3 and ω-6, based on the position of the first double bond relative to the ω end.

The anti-arrhythmic or cardioprotective effects of PUFAs, especially in patients with dyslipidemia, have been studied extensively (Rimm et al., 2018; Schmocker et al., 2018; Schunck et al., 2018; Zhang et al., 2018). Billman and others have shown that the omega-3 PUFA eicosapentaenoic acid prevented ischemia-induced ventricular fibrillation in a dog model of sudden cardiac death (Billman et al., 1999), suggesting modulation by PUFAs of cardiomyocyte electrical activity (Bogdanov et al., 1998; Xiao et al., 2004; Blondeau et al., 2007; Leaf, 2007; Moreno et al., 2012). Some of the molecular mechanisms that may underlie the cardioprotective effects of PUFAs include effects on channel gating (Elinder and Liin, 2017) and membrane properties (or electrostatics) (Borjesson et al., 2010; Borjesson and Elinder, 2011; Liin et al., 2015), and have been comprehensively reviewed elsewhere (Leaf et al., 2003).

If the relative composition of FFA content in the heart can influence inflammatory responses, and affect cardiac dysfunction, then dietary PUFAs may prevent pathological levels of pro-inflammatory cytokines (Wen et al., 2011; Oikonomou et al., 2018), and cardiac dysfunction in patients with metabolic disorders. Moreover, PUFAs have been shown to decrease secretion of TNF-α, IL-1β, and IL-6 through a pathway involving M2 anti-inflammatory macrophages (Lyons et al., 2016). However, the molecular partners involved are unknown, and therefore the mechanisms are poorly understood. Future studies are needed to investigate the differences between inflammatory pathways activated by the cardioprotective PUFAs and the generally more damaging SFFAs (M1 anti-inflammatory macrophages) (Lyons et al., 2016), and whether further differences would be seen with acute versus chronic activation of these pathways.

Saturated long chain FFAs, particularly palmitic acid (PA, 16:0), which is one of the predominant FFAs in epicardial fat (Iacobellis and Bianco, 2011), are considered to be a more prominent contributor to systemic lipotoxicity compared to long chain monounsaturated FFAs such as oleic acid (OA) (van der Lee et al., 2000; Listenberger et al., 2003; Kien et al., 2005). Previously we demonstrated that exogenous application of PA conjugated with bovine serum albumin (BSA) shortened atrial action potential (AP) duration (APD), measured in guinea pig atrial myocytes, while OA prolonged atrial APD (Aromolaran et al., 2016). Similarly, Anumonwo’s group found that a short-term exposure to the saturated stearic acid caused both structural and electrical remodeling of atrial myocytes isolated from sheep (O’Connell et al., 2015), consistent with pathological cardiomyocyte remodeling. Notably, the American Heart Association (AHA) has reported a significant association between dietary FFA and the pathogenesis of a variety of cardiovascular diseases (Krauss et al., 2000). The 2013 AHA/American College of Cardiology (ACC) Guideline on Lifestyle Management to Reduce Cardiovascular Risk recommends that patients with elevated low-density lipoprotein (LDL)- cholesterol decrease the intake of dietary saturated fat to 5–6% of the total daily caloric intake (Eckel et al., 2014).

Despite these guidelines, diet-related diseases such as obesity, T2D and insulin resistance and associated cardiac dysfunction (Kien et al., 2005; Ashrafi et al., 2017; Valli et al., 2017; Sanchez et al., 2018) are still widespread. This suggests that other mechanisms or pathways are involved, such as inflammation and cytokine release, which are activated by SFFAs. Accordingly, the anti-inflammatory effects of current therapeutic interventions (statins or APOA1) (Goonasekara et al., 2010; Shapiro and Fazio, 2016) are promising and are likely to inform future studies.

The mechanisms of FFA toxicity are complex, involving multiple combinations of distinct signaling pathways (Rennison and Van Wagoner, 2009; Park and Goldberg, 2012), suggesting the need to expand our understanding of how the interplay of these pathways lead to a diseased state. In this review we provide insights on the relatively unexplored interplay between cardiac lipotoxicity (mediated by SFFAs) and inflammatory pathways (macrophages, toll-like receptors, proinflammatory cytokines) that impair ion channel function, leading to cardiac electrical activity and conduction abnormalities. Our expectation is that understanding the cardiac-specific inflammatory pathways may facilitate the design and rational development of effective therapeutic interventions. These mechanisms are discussed below.

### Saturated Free-Fatty Acids and Inflammation

Over the past 40 years we have been able to establish a role for SFFAs in inflammation (Ajuwon and Spurlock, 2005; Bradley et al., 2008; Bunn et al., 2010; Guzzardi and Iozzo, 2011; Huang et al., 2012; Wang et al., 2013; Schilling et al., 2013; Haffar et al., 2015). Cardiac or systemic inflammation (or dysregulation of the innate immune system) is thought to be a function of the body’s non-specific response to injury (Pohl and Benseler, 2013). In obese and diabetic patients, the increased rates of infection and poor wound healing have been associated with immune cell dysfunction (Joshi et al., 1999; Ferrucci and Fabbri, 2018; Frydrych et al., 2018; Jin et al., 2018; van Niekerk and Engelbrecht, 2018). Importantly, macrophages are associated with heightened immune responses to infectious pathogens and tissue damage in diabetes (Mirza et al., 2009; Mirza and Koh, 2011; Das et al., 2018; Liu et al., 2018) and obesity (Lopez-Pascual et al., 2018; Ramos Muniz et al., 2018; Ding et al., 2018).

**FIGURE 1:**
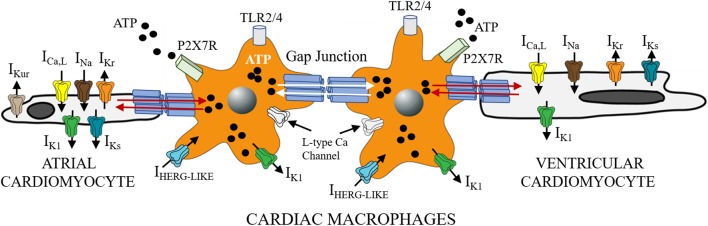
Functional crosstalk between macrophages and cardiomyocytes. The schematic shows functional coupling between macrophages and cardiomyocytes through gap junctional channels. Small molecules including ATP are exchanged between cells with implications for inflammatory response. Macrophages have been shown to express ion channels that are similar to depolarizing (*I_Ca_*, *I_Na_*) and repolarizing mechanisms in heart (*I_Kr_*, *I_Ks_*, and *I*_*K*1_). Previously Van Wagoner and others (Das et al., 2009), reported functional expression of Ca_v_1.2 channels in macrophages, that could be blocked by known Ca channel blockers (amlodipine and verapamil), but were voltage-independent. Macrophages also express I_HERG_-like currents, that are unrelated to cardiac *I_Kr_* (Pennefather et al., 1998; Zhou et al., 1998), and inwardly rectifying K channels (Moreno et al., 2013; Kan et al., 2016). What role, if any do the expression of these channels play in mediating the effects of SFFAs on cardiac electrical activity? Despite the lack of clarity, macrophages are likely to influence cardiac electrical function to help maintain homeostatic control. This influence could be via direct (TL4R-cytokine release) or indirect (ATP-P2X7R) signaling pathways. Distinguishing between multiple pathways is likely to further illuminate the role of cardiac macrophages in inflammation and the pathogenesis of lipotoxic cardiomyopathy.

Given that FFAs are important adipocyte-derived mediators of macrophage related inflammation (Suganami et al., 2005) and macrophages can infiltrate and/or directly couple with cardiomyocytes (Figure 1; Hulsmans et al., 2016; Sager et al., 2016), then macrophages may be important mediators of SFFA effects on cardiac electrical remodeling (Wang et al., 2017, 2018). Distinct voltage-dependent *K* channels (VGKC) including ether-á-go-go related gene 1 (ERG1), (Dong et al., 2013), inward rectifier (Vicente et al., 2003; Moreno et al., 2013), and shaker-related or K_v_1.3 (Vicente et al., 2003; Park et al., 2006; Villalonga et al., 2010) are found in macrophages. In addition to their role in controlling cardiac repolarization and resting membrane potential (Grandi et al., 2017; Jeevaratnam et al., 2018), VGKC are also involved in macrophage functions (activation, migration, proliferation) (Vicente et al., 2003). Because altered functions of macrophages could have important implications for proinflammatory cytokine release and arrhythmias, future studies will have to characterize the biophysical effects of VGKC localized to macrophages more precisely and determine whether impaired cardiac electrical activity includes altered macrophage ion channel function. For example, does the modulatory signaling pathways (including protein kinases, protein phosphatases, trafficking, anchoring proteins, and posttranslational modifications) that regulate cardiac VGKC subunit expression also regulate these channels in macrophages? These studies are a prerequisite for development of new therapeutics which target inflammatory pathways in lipotoxic-related disorders.

SFFAs have been shown to stimulate an inflammatory response by acting on TLRs present on macrophages (Figure 1). TLRs are pattern recognition receptors that play an important role in the body’s innate immune response (Takeda et al., 2003; Schilling et al., 2013). Thus, if inflammation is a hallmark of lipotoxicity (Ertunc and Hotamisligil, 2016; Ralston et al., 2017), then the role of SFFAs as an activator of TLRs must be understood because they initiate inflammation.

### Saturated Free-Fatty Acids and Toll-Like Receptor Signaling

**FIGURE 2:**
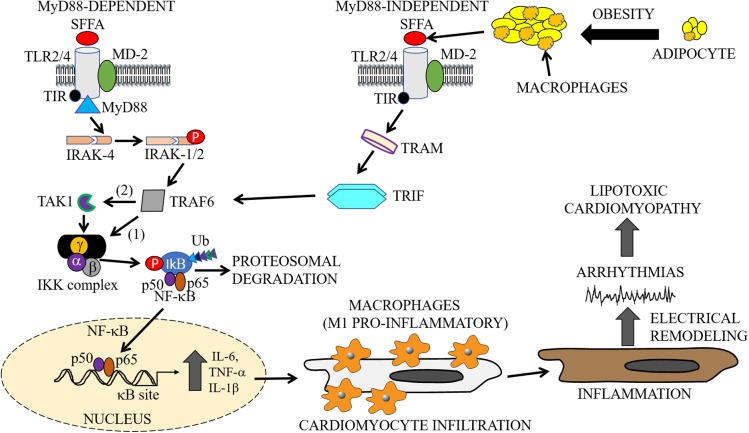
Cardiac and macrophage inflammatory pathways. In metabolic disorders, enlargement of adipocytes contributes to the activation of macrophages and release of SFFAs through lipolysis (Watanabe et al., 2013). The exposure of cells to increased levels of these inflammatory molecules leads to the activation of inflammatory pathways. The cartoon representation shows the activation of the TLR signaling pathways (MyD88-dependent and -independent), by SFFAs in both macrophages and cardiomyocytes. Infiltration of cardiomyocytes by M1 pro-inflammatory macrophages may lead to cardiac electrical dysfunction, cause arrythmias leading to cardiomyopathies of metabolic disorders. The MyD88-dependent pathway involves sequentially, activation of IRAK4, phosphorylation of IRAK1/2, and TRAF6, followed by activation of IKK and NF-κB, and the subsequent production or secretion of pro-inflammatory cytokines (IL-6, TNF-α, IL-1β). The MyD88-independent pathway also involves the secretion of pro-inflammatory cytokines through activation of TRAF6 by TRAM/TRIF. SFFA, Saturated free-fatty acid; TLR, Toll-like receptor; MD-2, myeloid differentiation protein-2; TIR, Toll/interleukin-1 receptor; TRIF, TIR-domain-containing adapter-inducing interferon-β; TRAM, TRIF-related adapter molecule; TRAF, tumor necrosis factor receptor-associated factor; IRAK, interleukin-1 receptor-associated kinase; TAK, transforming growth factor β activated kinase; IKK, inhibitory kappa B alpha kinase; NF-κB, nuclear factor-kappa B; Ub, ubiquitin.

Structurally, TLRs are characterized by three distinct domains, namely: (1) an extracellular leucine-rich repeat (LRR) domain, which is required and necessary for ligand binding and the recognition of pathogen-associated molecular patterns (PAMPs); (2) a transmembrane domain important for receptor localization to the surface (TLR1, TLR2, TLR4, TLR5, TLR6) and intracellular membranes (TLR3, TLR7, TLR8, TLR9); 3) a cytoplasmic conserved toll/interleukin-1 receptor (TIR) domain, which plays a role in the activation of NF-κB, and cytokine secretion (Figure 2; Fuentes-Antras et al., 2014). TLR2 and TLR4 have been widely studied and have both been shown to play a role in the pathogenesis of lipid disorders like atherosclerotic cardiovascular disease (Curtiss and Tobias, 2009), insulin resistance (Devaraj et al., 2008; Wong and Wen, 2008; Kim et al., 2010; Dong et al., 2012), and obesity (Kim et al., 2007; Ghanim et al., 2017).

SFFAs have been shown to promote both TLR4-dependent and TLR2-dependent signaling in multiple cell models. For example, Lee et al. (2001, 2004), using RAW 264.7 macrophages, demonstrated that the saturated lauric acid (C12:0), signaled *via*: (1) TLR4-myeloid differentiation primary response 88 (MyD88) to activate NF-κB; (2) TLR4-Toll-IL-1 receptor (TIR)-domain-containing adapter-inducing interferon-β (TRIF), leading to the activation of interferon-stimulated regulatory element. Furthermore, endogenous SFFAs released from adipocytes have also been shown to activate co-cultured macrophages *via* TLR4 (Suganami et al., 2007), demonstrating an important crosstalk in adipose tissue, with implications for lipotoxicity and cardiac electrical remodeling. Similarly, TLR2 has been shown to mediate palmitate-induced insulin resistance in C2C12 myoblasts (Senn, 2006). Endothelial overexpression of TLR2 led to early development of atherosclerotic processes in the aorta of LDLr^-/-^ mice (Mullick et al., 2008), while the downregulation of TLR2 protected against atherosclerosis in LDLr^-/-^ mice (Mullick et al., 2005). Others have also shown similar effects of TLR2-deficiency in apoE^-/-^ mice (Liu et al., 2008; Madan and Amar, 2008). Reduced TLR4 expression and function protected against insulin resistance in a mouse model of systemic lipid infusion (Shi et al., 2006), demonstrating a role for TLRs in lipotoxic disorders (Bashir et al., 2016; Shen et al., 2018). Flier and colleagues also found that female C57BL/6 mice lacking TLR4 and fed a high fat diet developed increased obesity, but are partially protected from insulin resistance through a mechanism involving reduced inflammatory gene expression (including IL-6) (Shi et al., 2006).

T2 diabetic mice with mutated TLR4 prevented endothelial cell dysfunction, hyperglycemia and hypertension when compared with wild-type. These effects were largely due to suppression of oxidative stress signaling molecules nicotinamide adenine dinucleotide phosphate (NADPH) oxidase 1 and 4 (Liang et al., 2013). Furthermore, mice lacking either TLR4 or its downstream adapter protein MyD88 are protected against atherosclerosis (Bjorkbacka et al., 2004; Michelsen et al., 2004; Ding et al., 2012). In agreement, humans with TLR4 mutations, which lead to reduced receptor signaling and depressed inflammatory response, are also less susceptible to atherosclerosis (Arbour et al., 2000; Kiechl et al., 2002; Lin et al., 2012). Schwartz’s group (Kim et al., 2007) also found that thoracic aortic samples from TLR4 knockout mice (TLR4^-/-^) fed a high-fat diet did not develop vascular inflammation or insulin resistance.

TLR4 are also expressed in normal myocardium (Vaez et al., 2016), suggesting that it may play a role in cardiac function. Moreover, increased TLR4 expression has been reported in failing myocardium (Frantz et al., 1999), and isolated cardiomyocytes from humans and animal models of different cardiomyopathies (Dange et al., 2014; Avlas et al., 2015; Liu et al., 2015), including myocarditis (Timmers et al., 2008). These findings provide strong hints that blocking TLR4 may be a promising cardioprotective target in patients. Whether and how effective modulation of the TLR4 pathway is as a therapeutic target is yet to be determined.

### NF-κB Activation Signaling Pathways

Activated TLR4 and myeloid differentiation protein-2 (MD-2) signals through both the MyD88-dependent and MyD88-independent pathways (Figure 2; Lee et al., 2003; Gao et al., 2017). The MyD88-dependent pathway is initially triggered by the recruitment of the TIR domain containing adaptor protein (or TIRAP), an adaptor between the TIR domain of TLR4 and MyD88 (Sakaguchi et al., 2011). This is followed by activation of the interleukin-1 receptor-associated kinase (IRAK) 4 family of protein kinases which induces IRAK1/2 phosphorylation and the subsequent phosphorylation of TNF Receptor Associated Factor 6 (TRAF6) (Vilahur and Badimon, 2014).

However, with the MyD88-independent pathway, the TLR4 pathway is activated through a TIR-containing adapter molecule 1 (TRIF)-related adaptor molecule TRAM. This allows the recruitment of TRIF and subsequent activation of TRAF6 (Figure 2). TRAF6, either directly, or *via* thylakoid arabidopsis kinase (TAK) 1, stimulates the inhibitor of nuclear factor-κB kinase (IKK) complex, which promotes phosphorylation of the inhibitor of NF-κB (IkB). In the resting state of a cell, IkB exists in a complex with NF-κB, which limits its spatial localization to cytosol. Phosphorylated IkB is ubiquitinated, dissociates from NF-κB, and is then subjected to proteasomal degradation. Free NF-κB (p50/p65 heterodimer) translocate to the nucleus and binds to κB sites in the promoter regions of genes involved in the secretion of inflammatory cytokines which includes TNF-α and IL-6 (Youssef-Elabd et al., 2012). Furthermore, the p50/p65 heterodimer may undergo a series of cellular protein modifications including phosphorylation, acetylation, and methylation with implications for a finely-tuned regulation of transcriptional activity of pro-inflammatory cytokines. Moreover, NF-κB transcriptional activity may also be regulated by secreted pro-inflammatory cytokines through a positive and/or negative feedback mechanism depending on the pathological state of the cell.

NF-κB-mediated cytokine release and inflammation may also be augmented through adenosine triphosphate (ATP) *via* purinergic signaling (Figure 1; Dosch et al., 2018; Lee et al., 2018). In pathological conditions, ATP from cells pass through hemichannels to activate purinergic receptors, leading to an amplification of inflammation (Mezzaroma et al., 2011). Moreover inhibition of the ATP receptor P2X7 prevented cardiac dysfunction in a mouse model of acute myocardial infarction (Mezzaroma et al., 2011) and LPS-primed naive rats (Yin et al., 2017). P2X7 deficiency inhibited inflammasome activation and reduced atherosclerosis in P2X7^-/-^ mice (Stachon et al., 2017). Therefore, activation of connexins and P2X7 receptors (P2X7R) signaling pathways represent emerging targets that can be explored in lipotoxic cardiomyopathies.

### NF-κB and Cardiomyocyte Remodeling

The role of NF-κB as a key transcription factor critical for regulation of cardiac inflammatory signaling pathways strongly suggests its involvement in cardiac remodeling leading to pathogenesis of heart failure (Zhou et al., 2009; Santos et al., 2010; Schreiber et al., 2011). Previous reports have shown that NF-κB is activated in the failing human heart (Wong et al., 1998; Gupta and Sen, 2005; Kawamura et al., 2005). Further, *in vitro* studies have also demonstrated that activation of NF-κB plays a role in hypertrophic growth of primary rat neonatal ventricular cardiomyocytes in response to angiotensin II, phenylephrine, and endothelin-1 (Purcell et al., 2001). Recent studies have also demonstrated that NF-κB plays a partial role in the depression of the fast transient outward K current (*I_to,f_*) caused by chronic β-adrenergic receptor stimulation in cultured neonatal rat ventricular myocytes (Panama et al., 2011, 2016). In these studies NF-κB effects were attributed to a downregulation of the pore-forming (K_v_4.3) and regulatory/auxiliary (KChIP2) subunits of *I_to,f_* (Panama et al., 2011). The functional consequence of NF-κB modulation may be due to transmural changes in *I_to,f_* in the ventricles and possibly the atria. These effects further highlight an emerging role for NF-κB as a substrate for cardiac dysfunction in lipotoxicity.

In a study by Kawamura and others, the blockade of NF-κB did not ameliorate myocardial inflammation, but significantly improved cardiac function and survival in a transgenic mice model with cardiac overexpression of TNF-α (Kawamura et al., 2005). Recently, a similar mechanism has been proposed for TRAF1, an inhibitory adapter of TLRs (Anto Michel et al., 2018). It was found that downregulation of macrophage-resident TRAF1 induced increased expression of inflammatory genes and protected against metabolic dysfunction in mice. Therefore, it is possible that activation of the non-canonical NF-κB/TRAF1 pathway (Choudhary et al., 2013), may at least, in part, explain the effects of TNF-α described by Sunagawa’ group (Kawamura et al., 2005).

The studies of Wolf and others also showed a convincing correlation between higher TRAF1 expression, body mass index, and fasting plasma lipid in patients with severe metabolic syndrome (Anto Michel et al., 2018). Albeit interesting, the implications of these findings on cardiac function were not investigated. To reconcile existing knowledge of the relationship between metabolic disorders, inflammation and heart failure, we will need additional studies related to the spatial and temporal effects of TRAF1, inflammatory responses and effects on cardiac remodeling.

Nonetheless, we speculate that NF-κB activation may contribute to cardiac dysfunction independent of macrophage-related inflammation. Therefore, blockade of NF-κB leading to cardiac electrical remodeling may be a novel therapeutic strategy for cardiac diseases worth investigating. However, little is known about the effects of NF-κB inhibition in patients with metabolic disorders or any animal models of cardiac lipotoxicity and metabolic disorders including T2D, insulin resistance, and cardiac inflammation.

### Saturated Free-Fatty Acid, NF-κB, and Proinflammatory Cytokines

**FIGURE 3:**
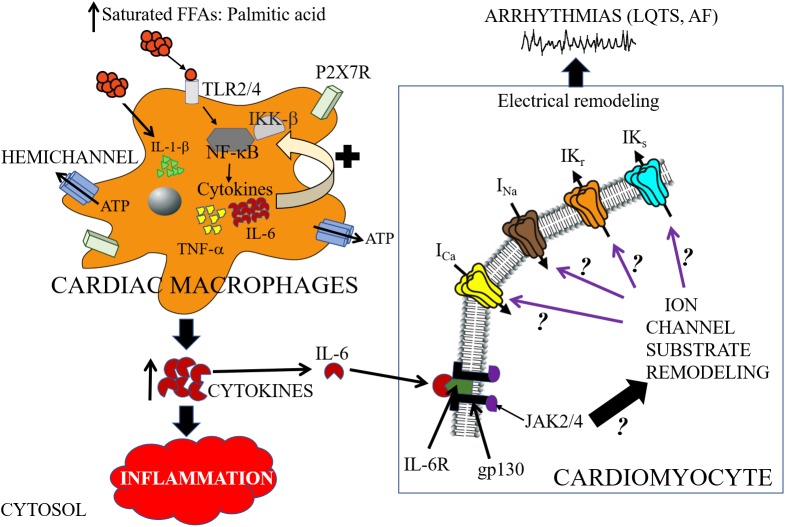
Cartoon depiction illustrating potential saturated free-fatty acid mechanisms that may underlie arrhythmogenic events. High-fat diet induce elevations of saturated free-fatty acids (SFFAs) leading to increased expression of proinflammatory cytokines in macrophages. Cardiac macrophages sense high concentrations of SFFAs through the toll-like receptor 2 (TLR2) and/or toll-like receptor 4 (TLR4) which induces expression of inflammatory target genes including tumor necrosis factor (TNF)-α and interleukin-6 (IL-6), by regulating the activities of nuclear factor-κB (NF-κB) (Youssef-Elabd et al., 2012). SFFAs also induce interleukin-1β secretion via the NLRP3 inflammasome in macrophages, which is independent of the TLR4 pathway (Henao-Mejia et al., 2012). Hemichannels control the exchange of small molecules including ATP (Willebrords et al., 2016). The released ATP binds to the cell-surface purinergic receptors P2X7R, and can control inflammation by regulating the release of pro-inflammatory cytokines (including IL-1β) (Gicquel et al., 2017). There is a paucity of lipid studies involving the IL-6 signaling pathway, and therefore the molecular mechanisms of IL-6 modulation of cardiac function is poorly understood. In cardiomyocytes, IL-6 mediates its effect through its classical pathway. IL-6 binds to the glycoprotein 80 transmembrane IL-6 receptor (IL-6R) (Yamasaki et al., 1988), which leads to dimerization and activation of the signal transducing protein of glycoprotein 130 (gp130) and its downstream effector signaling molecules Janus kinase (JAK) 2/4 (Ihle, 1995; Heinrich et al., 2003; Drucker et al., 2010). We hypothesize that SFFA mediated cardiac electrical remodeling that increase vulnerability to arrythmias may occur through SFFAs/TLR/NF-kB-IL-6 mediated altered gene and protein expression of ion channel subunits, trafficking and/or gating defects. Distinguishing among these signaling pathways is likely to provide mechanistic insights that will influence considerations of anti-cytokine therapy for the management of metabolic disorders and arrythmias. The black ? indicates that whether or how activation of JAK2/4 leads to remodeling of ion channel molecular partners is poorly understood. The black? (associated with the purple arrows), represents an unresolved role for IL-6 modulation of major cardiac ion channels (*I_Ca_*, *I_Na_*, *I_Kr_*, and *I_Ks_*) in lipotoxicity and pathogenesis of arrhythmias including long QT syndrome (LQTS) and atrial fibrillation (AF).

A major signaling pathway for SFFA-mediated inflammatory response and cardiac electrical dysfunction may involve the following steps: (1) infiltration of cardiac macrophages into cardiomyocytes (2) increased functional expression and activation of TLR; (3) activation of NF-κB; (4) altered secretion of pro-inflammatory cytokines; and (4) ion channel remodeling (Figure 3). Recently the significance of this pathway was demonstrated in a LIPGENE cohort study (Cruz-Teno et al., 2012), wherein NF-κB was found to regulate TNF-α release during postprandial period (Lefebvre and Scheen, 1998). Because the activation of this pathway may also depend on the amount and type of dietary fat, the potential of this pathway as an intervention strategy in overweight and obese patients with cardiac dysfunction deserves further investigation.

The TNF-α signaling pathway and its role in cardiac ion channel remodeling leading to cardiac dysfunction in animal models has been extensively studied (London et al., 2003; Wang et al., 2004; Hatada et al., 2006; Kawada et al., 2006; Petkova-Kirova et al., 2006; Fernandez-Velasco et al., 2007; Grandy and Fiset, 2009). However, the functional consequence of the relative contribution of each one of the steps (SFFAs/TLR4/NF-κB) involved in TNF-α production and its subsequent regulation of cardiac function in relevant animal models of cardiac lipotoxicity is poorly understood. In contrast, it is known that the stimulatory effects of distinct SFFAs (lauric, palmitic and stearic acids) cause increased IL-6 functional expression in macrophages *via* TLR4 activation (Shi et al., 2006). Despite the role of IL-6 as a cardiovascular risk indicator (Lazzerini et al., 2017a), its function as modulator of cardiac electrical remodeling with implications for the pathogenesis of arrythmias is only beginning to be understood. In this review, we focus on the pathophysiology of the IL-6 signaling pathway and how its modulation may reveal crucial insights that will inform future lipid studies (Table 1).

**Table 1:** Correlation between IL-6, cardiac ion channel expression, and contractile function in arrhythmias.

IL-6	Cardiomyopathy	Model	Ion channel and cardiomyocyte contractile function	Reference
↑	1. LQTS, 2. Ventricular arrythmias	Human	NR	Streitner et al., 2007; Lazzerini et al., 2016, 2017b
1000 U/ml (5 min)	NR	Adult female guinea pig ventricular myocytes	↓*I_Ca_*, ?*I_Na_*, ?*I_Kr_*, ?*I_Ks_*	Sugishita et al., 1999
			↓contractility	
			↓ [Ca^2+^]_i_ transient	
400 pg/ml (1–3 h)	NR	Adult Rat (SD), ventricular myocytes.	↔ Cardiac contraction	Maass et al., 2002
10 ng/ml (2–24 h)	NR	Adult Rat (SD), ventricular myocytes	Negative inotropy	Yu et al., 2005
			↓ Cell shortening	
			↔*I_Ca_*, ?*I_Na_*, ?*I_Kr_*, ?*I_Ks_*	
			↓SR Ca content	
			↓Phosphorylation of Phospholamban	
			↓SR Ca uptake	
			↓ Postrest potentiation and caffeine response	
50 Units/ml (6 h)	NR	Rat neonatal (1–2 day old; Wistar), cultured ventricular myocytes	↓ SERCA mRNA	Tanaka et al., 2004
10 ng/ml (48 h)	NR	Rat neonatal (1–2 day old), cultured ventricular myocytes	↓ SERCA2 mRNA	Villegas et al., 2000
			↓ SERCA2 protein	
20 ng/ml (20–40 min)	NR	Mice ventricular myocytes	↑ *I_Ca,L_*, ?*I_Na_*, ?*I_Kr_*, ?*I_Ks_*	Hagiwara et al., 2007
			↑ [Ca^2+^]_i_ transient	
			↑ APD	
1000 U/ml (30 min)	NR	Chick embryo cultured ventricular myocytes	Negative inotropy	Kinugawa et al., 1994
			↓Peak systolic [Ca^2+^]_i_	
			↓Cell contraction	
0.5–3000 ng/ml (10–30 min)	NR	Rat neonatal (2 day-old, Lewis) cultured ventricular myocytes	↔ Cell shortening	Kumar et al., 1996
0.1–3 ng/ml (2–5 min)	NR	Hamster papillary muscle	Negative inotropy	Finkel et al., 1992, 1993
↑	1. AF	Human	NR	Conway et al., 2004; Marin et al., 2004; Psychari et al., 2005; Boos et al., 2007; Fujiki et al., 2007; Gedikli et al., 2007; Ucar et al., 2007; Marcus et al., 2008, 2010; Chen et al., 2009; Henningsen et al., 2009; Leftheriotis et al., 2009; Qu et al., 2009; Cheng et al., 2012; Wu et al., 2013; Aulin et al., 2015; Pudil et al., 2016
	2. Atrial flutter			
	3. Atrioventricular nodal reentry tachycardia			
0.1–3 ng/ml (2–5 min)	NR	Human pectinate muscle (Atria)	NR	Finkel et al., 1993
↑	AF	Mice	NR	Ozcan et al., 2015


*↑, increased; ↓, decreased; ↔, no change; NR, not reported; SD, Sprague Dawley; WR, Wistar Rats.*

### Molecular Mechanisms of Interleukin-6 Signaling

IL-6 is a pleiotropic cytokine that is involved in a variety of biological effects that occur in cells of the immune system and also cardiomyocytes in response to injury (Ancey et al., 2002; Yang et al., 2004). IL-6 mediates its effects either through its membrane-bound receptor, IL-6R alpha (α) subunit (classical signaling) or the soluble receptor (sIL-6R) (Taga and Kishimoto, 1997; Fontes et al., 2015), in complex with the signal transduction protein glycoprotein 130 (gp130) leading to the activation of the janus kinase (JAK)-related signaling pathways (Akira et al., 1990, 1994; Naka et al., 1997; Taga and Kishimoto, 1997; Figure 3).

### Interleukin-6 Signaling and Propensity for Cardiac Arrhythmias

Cardiac arrhythmias are irregular variations from the normal cardiac sinus rhythm due to conduction abnormalities, or electrical impulses in the heart. It is well established that the abnormal electrical activity that predispose to arrhythmias is commonly due to dysfunction and/or structural disruption of the electrical conduction system of the heart and can be classified based on their pathogenesis. In particular, the underlying molecular mechanisms of IL-6 in the pathogenesis of ventricular and supraventricular arrhythmias are poorly understood. In this review we highlight current reports of IL-6 involvement in the pathogenesis of cardiac dysfunctions associated with increased vulnerability to fatal ventricular arrhythmias (long QT syndrome or LQTS) (Schwartz et al., 2009; Beitland et al., 2014) or increased morbidity (atrial fibrillation or AF) and mortality (Table 1). Our hope is that studies that distinguish between the molecular mechanisms of IL-6 contribution to LQTS and AF may reveal important mechanistic insights that will inform targeted therapeutic interventions that will be beneficial to all patients and help improve quality of life.

#### Interleukin-6 and Long QT Syndrome

Previous studies have shown that circulating IL-6 levels are elevated in patients with autoimmune diseases (Adlan et al., 2015; Lazzerini et al., 2015a,c, 2017a) and are associated with the prolongation of corrected QT (QT_c_) or LQTS, a serious condition which increases vulnerability to fatal arrhythmias including *Torsades de Pointes* (*TdP*). These results were obtained by accumulating data from patients with myo/endocarditis (Ukena et al., 2011; Lazzerini et al., 2015b) and systemic autoimmune diseases, particularly rheumatoid arthritis (Lazzerini et al., 2014, 2017a; Adlan et al., 2015) and connective tissue disease (Lazzerini et al., 2015c). There have also been reports of an association between elevated serum IL-6 concentrations and increased susceptibility to spontaneous ventricular tachyarrhythmia in patients with coronary artery disease (Streitner et al., 2007). IL-6R expression is also upregulated in heart failure and therefore underscores an additional therapeutic role of IL-6R blockers in lipotoxic cardiomyopathies.

#### Interleukin-6 and Atrial Fibrillation

Altered IL-6 functional expression is also a common feature of supraventricular arrythmias including AF (Marcus et al., 2008; Pan et al., 2018), leading to higher risks of death and cardiovascular events in AF patients (Aulin et al., 2015). Moreover, increased IL-6 levels have been attributed to persistent inflammation in atrial myocardium (Stein et al., 2008; Amdur et al., 2016; Pan et al., 2018) and further supports the idea of an important functional link to supraventricular arrhythmias. Short-term (2 weeks) administration of the TLR4 agonist, LPS, induced NF-κB activation, increased IL-6 concentration (in plasma and right atrium), and increased vulnerability to AF in a canine model of systemic inflammation. The intimate structural relationship between SFFAs and LPS (Hwang et al., 2016) would imply that similar mechanisms may underlie vulnerability to arrhythmogenic events mediated by SFFAs. These mechanisms warrant further analysis in cardiomyocytes and animal models.

Similar to LPS, SFFA activation of TLR4 increases circulating IL-6 levels in macrophages (Bosisio et al., 2002; Castelli et al., 2015). Therefore, considering the idea of reported functional expression of TLR4 in cardiomyocytes (Frantz et al., 1999; Avlas et al., 2011; Zhao et al., 2016a; Chimenti et al., 2017; Jiang and Qu, 2017; Jiang et al., 2018) and the notion that cardiomyocytes secrete IL-6 (Ancey et al., 2002; Maass et al., 2002), it will also be important to determine the temporal and relative contribution of these distinct pathways to impaired cardiac dysfunction mediated by the direct effects of SFFAs on cardiac function (Haim et al., 2010; O’Connell et al., 2015; Aromolaran et al., 2016). The data is likely to reveal new targets that may be relevant to therapeutic responses in patients with metabolic disorders. This can be easily advanced by lipid studies in relevant animal and translational models of AF progression.

The growing evidence of a critical link between inflammation and arrhythmias provide strong initial clues as to the potential effects of SFFAs on ion channels through inflammatory cytokine signaling pathways leading to altered APD and QT_c_ interval. Therefore, it would be interesting to delineate the functional interplay among individual steps in the SFFAs/TLR4/NF-κB/cytokine pathway leading to ion channel remodeling.

### Ion Channels and Cardiomyocyte Electrical Activity

The electrical activity of the human heart is controlled by the coordinated action of ion channels localized to distinct compartments (surface sarcolemma, t-tubular system, intercalated disk), within the myocardium. The temporal and biophysical properties of individual ionic channels allow the exchange of ions between distinct compartments and are responsible for generation of an AP in individual cardiomyocytes. The normal cardiac AP is defined by 5 distinct phases, namely: phase 0 or phase of rapid depolarization, due to a large inward Na current, *I_Na_* followed by currents due to voltage-gated L-type Ca (*I_Ca,L_*) and the sodium-calcium exchanger (*I_NCX_*) channels (Bers and Despa, 2009); phase 1 or phase of early repolarization controlled by the transient outward K current, *I_to_*; phase 2 or plateau phase accomplished by a balance between the depolarizing Ca current (*I_Ca,L_*) and the repolarizing rapidly (*I_Kr_*) and slowly (*I_Ks_*) activating component of the delayed rectifier K currents. The atria specific ultra-rapidly (*I_Kur_*) activating delayed rectifier K current controls repolarization (Tian et al., 2006) and underlies the triangular signature of the atrial AP (Ford et al., 2013); phase 3 (or phase of final repolarization) represents the inactivation of *I_Ca,L_* and is therefore predominantly regulated by the delayed rectifier K currents and the inwardly rectifying K current (*I*_*K*1_) (Varro et al., 1993). While extensive reviews about the pathophysiology of major cardiac ionic channels have recently been published (Aromolaran and Boutjdir, 2017; Dan and Dobrev, 2018; Heijman et al., 2018; Rahm et al., 2018), the importance of ion channel function to normal cardiac rhythm is exemplified in a variety of inherited and acquired pathological conditions. For example, in LQTS decreases in outward currents (Aromolaran et al., 2014; Puckerin et al., 2016) or increases in depolarizing mechanisms (Wehrens et al., 2003; Fredj et al., 2006; Cheng et al., 2011; Hsiao et al., 2013), predispose to fatal ventricular arrhythmias (such as *TdP*) (El-Sherif et al., 2017; Muser et al., 2018; Wit, 2018) and sudden cardiac death (Anderson et al., 2019; Liu et al., 2018).

In the atria, remodeled biophysical properties of ionic channels (Aromolaran et al., 2016) serve as substrates for AF, including accelerated repolarization, spatial and temporal AP instabilities, atrial refractoriness, early and delayed afterdepolarizations, ectopic firing, and single/multiple wave re-entrant mechanisms (Nattel and Dobrev, 2017; Dan and Dobrev, 2018; Heijman et al., 2018). Collectively these observations reveal potential targets for modulation by SFFA pathways.

Our recently published data suggest that IL-6 may be a critical inflammatory signaling molecule contributing to *TdP* in patients with rheumatoid arthritis (Lazzerini et al., 2017b). This finding identifies IL-6 as a potential therapeutic target in arrhythmias. In the following sections we highlight the modulation by IL-6 of *I_Na_*, *I_Ca,L_*, and *I_K_* currents critically involved in cardiac instabilities, that ultimately predisposes to lipotoxic cardiomyopathies.

## Molecular Remodeling of Cardiac Ion Channels by Interleukin-6

### Depolarizing Na Current (I*_Na_*)

The modulation of *I_Na_* by IL-6 in cardiomyocytes is poorly understood; however, there is evidence for modulation of *I_Na_* by other pro-inflammatory cytokines in other cell systems. The pro-inflammatory cytokine IL-2, which is also associated with arrhythmias, (Rizos et al., 2007) has been shown to increase the transcriptional levels of *SCN3B* leading to increased peak *I_Na_* density in Hela and HL-1 cells (Zhao et al., 2016b) and suggests a role for cytokine modulation of *I_Na_* functional expression in lipotoxicity. From the perspective of cardiac electrical remodeling, cytokine-mediated increases in *I_Na_* density will be expected to delay cardiac repolarization leading to prolongation of the QT interval. Future IL-6 studies in native atrial and ventricular cardiomyocytes and animal models of lipotoxicity are critical for fundamental insights into the functional consequence of IL-6 modulation of Na current in metabolic disease-related arrhythmias.

### L-Type Ca Channels (I*_Ca,L_*)

Unlike *I_Na_*, IL-6 has been shown to regulate *I_Ca,L_*_,_ density. Hagiwara and others (Hagiwara et al., 2007) demonstrated that acute (30 min) exposure to IL-6 and sIL-6R significantly increased *I_Ca,L_* density in mouse ventricular myocytes in line with a role for IL-6 in LQTS. In guinea pig ventricular myocytes acute (5 min) exposure to IL-6 had no effect on *I_Ca,L_*, but reversed the increased *I_Ca,L_* due to sympathetic stimulation with isoproterenol (Sugishita et al., 1999). By contrast, chronic (2 h) exposure to IL-6 alone had no effect on *I_Ca,L_* in adult rat ventricular myocytes (Yu et al., 2005) and therefore suggests temporal differences in IL-6 effects on channel function.

The Ca_v_1.2 channel subunit mediates *I_Ca,L_* current in both the atria and ventricles (Mancarella et al., 2008). Targeted deletion in mice has also demonstrated a functional role of the Ca_v_1.3 isoform in the pathogenesis of AF (Zhang et al., 2005; Mancarella et al., 2008; Lu et al., 2015; Sun et al., 2017). Therefore, it will be interesting to investigate the differential regulation of Ca_v_1.2/Ca_v_1.3 by IL-6. With expression of Ca_v_1.3 only in the atria we may be able to identify new pathways that could be targeted for arrhythmias in patients with metabolic disorders, without off-target ventricular effects.

In agreement with this notion, we have recently found that Ca_v_1.3 expression is significantly downregulated in the atria of high-fat diet induced obese guinea pigs, while Ca_v_1.2 expression remained essentially unchanged (Ademuyiwa S Aromolaran, personal communication, Biophysical meeting 2018, San Diego, CA, United States). Although the role of Ca_v_1.3 in inflammation remains to be defined, the data suggests that Ca_v_1.3 expression and regulation could be participating in arrhythmogenic responses to lipotoxicity.

IL-6 studies in mice ventricular myocytes showed that acute exposure (10 min) to IL-6 and sIL-6R significantly increased intracellular Ca transients (Hagiwara et al., 2007). This suggests a role for IL-6 in mediating arrhythmias through modulation of Ca-handling proteins. Yu et al. (2005) showed that IL-6 induced negative inotropy, decreased postrest potentiation, as well as responsiveness to the ryanodine receptor (RyR) agonist caffeine in adult rat ventricular myocytes. Similarly, acute exposure to IL-6 produced decreased peak systolic intracellular Ca concentration ([Ca^2+^]_i_) and cell shortening leading to a negative ionotropic effect in guinea pig ventricular myocytes despite a lack of effect on *I_Ca,L_* (Sugishita et al., 1999). Human recombinant IL-6 significantly decreased peak systolic [Ca^2+^]_i_ and the amplitude of cell contraction in cultured chick embryo ventricular myocytes (Kinugawa et al., 1994).

IL-6 may also directly regulate the activity of the sarcoplasmic reticulum Ca-ATPase (SERCA2). Previous reports showed that chronic (48 h) exposure of IL-6 caused a significant downregulation of SERCA2 gene and protein expression levels in cultured neonatal rat ventricular myocytes (Villegas et al., 2000), while a 6 h exposure to IL-6 significantly decreased SERCA2 expression in cultured rat ventricular myocytes (Tanaka et al., 2004), consistent with impaired sarcoplasmic reticular (SR) function and propensity for arrhythmias.

The regulation by IL-6 of intracellular Ca dynamics through modulation of RyRs or the inositol triphosphate receptor (IP_3_R) is currently unknown. In the context of metabolic diseases, altered IL-6 mechanisms that decrease *I_Ca,L_* density, reduce intracellular Ca transients and impair cardiac contractility are also likely to promote supraventricular arrhythmias (Mancarella et al., 2008). To our knowledge, the underlying molecular mechanisms of IL-6 modulation of Ca_v_1.3 and Ca handling proteins in atrial myocytes are poorly understood and therefore warrants future studies. Nevertheless, the outcome of IL-6 studies in ventricular myocytes demonstrate that IL-6-mediated altered Ca handling proteins and subsequent inhibition of the SR function may contribute to impaired cardiac electrical activities in lipotoxicity.

### The Delayed Rectifier K Current (I*_K_*)

Cardiac delayed rectifier K current, (or *I_K_*) contributes prominently to normal repolarization (Sanguinetti and Jurkiewicz, 1990). The molecular partners of *I_K_*_,_ namely: *I_Kr_* and *I_Ks_* determine spatial and temporal activation and modulation of repolarization by *I_K_* (Sanguinetti and Jurkiewicz, 1991; Aromolaran et al., 2014). Thus, inactivation and/or downregulation of *I_K_*, either due to congenital mutations (Aromolaran et al., 2014; Puckerin et al., 2016), or secondary to pathological disease states including diabetes (Eranti et al., 2016), obesity (Papaioannou et al., 2003), or drugs (Shenthar et al., 2017; Grouthier et al., 2018), will delay repolarization leading to LQTS. Recently, we demonstrated that both gating and trafficking defects underlie pathological decreases in *I_Kr_* and *I_Ks_* in heart (Aromolaran et al., 2014; Puckerin et al., 2016).

A previous report by Wang et al. (2004), showed TNF-α mediated depression of the human ether-à-go-go-related gene (or hERG) current density in human embryonic kidney (HEK293) cells. Further, TNF-α significantly reduced *I_Kr_* density and prolonged APD in canine ventricular myocytes, primarily though changes in reactive oxygen species (Wang et al., 2004). The effects of IL-6 on *I_Kr_* and *I_Ks_* currents as targets in its reported link to LQTS (Lazzerini et al., 2017a) is currently unknown. This is further complicated by a lack of clarity about the role of *I_K_* in AF. We have previously reported that the SFFA PA increased the densities of *I_Kr_* and *I_Ks_* currents in HEK293 cells and shortened atrial APD measured in adult guinea pig myocytes, in line with a role for *I_K_* in AF associated with metabolic disorders (Aromolaran et al., 2016). Therefore, considering the idea that SFFAs activate macrophages and promote increased expression of IL-6 (Shi et al., 2006; Figure), our expectation is that *I_K_* may also be an important target for IL-6 modulation in AF pathogenesis in patients with metabolic disorders.

## Future Directions

Metabolic disorders and cardiac arrhythmias are interlinked epidemics with significant implications for public health. One explanation for the lack of progress may be due to incomplete understanding of cardiac electrical remodeling initiated through systemic and/or localized effects of SFFAs. Delineating unappreciated SFFA pathways could represent the basis for gaining new molecular mechanistic insights with implications for prevention of arrhythmias. This notion could be highlighted by the paucity of lipid studies that incorporate modulation by cytokines of cardiac ion channels, notably *I_K_* (*I_Kr_* and *I_Ks_*) a prominent repolarizing mechanism in heart.

We have focused on the pro/anti-inflammatory cytokine IL-6 as an important target for future investigation. This premise is based on our recent finding that pathological alterations in IL-6 may underlie arrhythmic risk in *TdP* patients (Lazzerini et al., 2017a). This study suggests the potential of anti-cytokine therapy as a novel treatment option. If proven, this may represent the missing link that we need to develop safer and more effective interventions, especially in *TdP* patients unresponsive to conventional treatment. The implication of altered IL-6/IL-6R signaling for arrhythmias in patients with metabolic disorders is currently not clear. In this context it will be interesting to: (1) know the differential expression of IL-6R in cardiomyocytes and in different subtypes of macrophages (2) investigate sources, spatial and temporal IL-6 concentrations, (3) distinguish between modifying enzymes and signaling pathways in specific cardiac regions, (4) understand the role of resident cardiac macrophages in the interplay between different cells in adipose tissues and how this may be affected in metabolic disorders.

Furthermore, the implications of production of distinct FFAs (SFFAs, monounsaturated FFAs, polyunsaturated FFAs) and cytokines (IL-6, TNF-α, IL-1β), from different sites (adipose tissue depot, macrophages, fibroblasts, lymphocytes, neutrophils, and cardiomyocytes), would be an interesting area of investigation. These pathways could be defined in animal models of inflammation and lipotoxicity, without confounding co-morbidities. Moreover, studies of individual disease phenotypes will allow us to define the specificity and linkage between multiple signaling pathways. This is especially important for precision or personalized medicine and development of targeted therapeutics for distinct arrhythmogenic mechanisms in patients.

It is noteworthy that the translational impact of mechanisms defined in animal models (mice, rats, guinea pig, rabbits, sheep), may be limited by differences in genetic background. Therefore, with more studies utilizing state-of-the art approaches, including human induced pluripotent stem (hiPSC)-derived cells (cardiomyocytes, macrophages, fibroblasts) and CRISPR-Cas9, to assess cardiac mechanisms of lipotoxicity, we may be able to better understand unique phenotypes in patients and improve our efforts to develop basic patient-specific interventions.

## Conclusion

Studies have revealed that there is a pathological link between dietary SFFAs and cardiac dysfunction. The increasing epidemic of metabolic disorders (dyslipidemia, obesity, T2D, insulin resistance, hyperglycemia), suggests that vulnerability to fatal arrhythmias and sudden cardiac death will remain high in patients. Despite advances in prognostic approaches and therapeutics, it is becoming increasingly clear that significant adjustments of existing approaches are needed. Pro-inflammatory signaling pathways are beginning to emerge as substrates for sustained lipotoxic events. While pathological inflammatory substrates may impair the ability of the heart to compensate for lipotoxic cardiomyopathies, the complexity of individual and synergistic effects of pro-inflammatory cells/cardiomyocytes limits a holistic understanding of this coupling. Importantly, the interplay with mechanisms (ion channel modulation) that lead to electrophysiological remodeling that support sustained and fatal arrhythmias are poorly understood. Therefore, if we understand the basic mechanisms involved, we may be able to prevent the abnormalities that eventually predispose patients to heart failure. With the increasing advent of promising tools (Garg et al., 2018; Heijman et al., 2018), we are better equipped to identify novel therapeutic sites.

## Author Contributions

AA researched concepts, and edited and finalized the manuscript. MB finalized the manuscript. ASA obtained funding, conceived of, and wrote the manuscript.

## Conflict of Interest Statement

The authors declare that the research was conducted in the absence of any commercial or financial relationships that could be construed as a potential conflict of interest.

## Abbreviations

AF: Atrial fibrillation
LQTS: Long QT syndrome
QT_c_: QT interval corrected for heart rate
AP: Action potential
APD: Action potential duration
*I_Kr_*: Rapidly activating delayed rectifier K current
*I_Ks_*: Slowly activating delayed rectifier K current
*I_Na_*: Voltage-gated Sodium current
*I_Ca_,_L_*: Voltage-gated L-type Ca current
*I_to_*: Fast transient outward K current
hERG: Human ether-á-go-go-related gene
HEK: Human Embryonic Kidney
FFAs: Free-fatty acids
SFFAs: Saturated Free-fatty acids
RyR: Ryanodine receptors
IP_3_R: Inositol trisphosphate
SR: Sarcoplasmic reticulum
SERCA: Sarcoplasmic endoplasmic reticulum Ca-ATPase pump
PA: Palmitic acid
OA: Oleic acid
T2D: Type 2 diabetes
IL-6: Interleukin-6
TLR: Toll-like receptor
TNF-α: Tumor necrosis factor alpha
NF-κB: Nuclear factor kappa-light-chain-enhancer of activated B cells
TdP: *Torsades de Pointes;* TIR Toll/interleukin-1 receptor
TRIF: TIR-domain-containing adapter-inducing interferon-β
TRAM: TRIF-related adapter molecule
TRAF: tumor necrosis factor receptor-associated factor
TAK: transforming growth factor β activated kinase
IRAK: interleukin-1 receptor-associated kinase
IKK: inhibitory kappa B alpha kinase
MD-2: myeloid differentiation protein-2
Ub: ubiquitin

## References

[B1] Adlan A. M. Panoulas V. F. Smith J. P. Fisher J. P. Kitas G. D. (2015). Association between corrected QT interval and inflammatory cytokines in rheumatoid arthritis. *J. Rheumatol.* 42 421–428. 10.3899/jrheum.140861 25593223

[B2] Ajuwon K. M. Spurlock M. E. (2005). Palmitate activates the NF-kappaB transcription factor and induces IL-6 and TNFalpha expression in 3T3-L1 adipocytes. *J. Nutr.* 135 1841–1846. 10.1093/jn/135.8.1841 16046706

[B3] Akira S. Isshiki H. Sugita T. Tanabe O. Kinoshita S. Nishio Y. (1990). A nuclear factor for IL-6 expression (NF-IL6) is a member of a C/EBP family. *EMBO J.* 9 1897–1906. 10.1002/j.1460-2075.1990.tb08316.x 2112087 PMC551896

[B4] Akira S. Nishio Y. Inoue M. Wang X. J. Wei S. Matsusaka T. (1994). Molecular cloning of APRF, a novel IFN-stimulated gene factor 3 p91-related transcription factor involved in the gp130-mediated signaling pathway. *Cell* 77 63–71. 10.1016/0092-8674(94)90235-6 7512451

[B5] Altarejos J. Y. Taniguchi M. Clanachan A. S. Lopaschuk G. D. (2005). Myocardial ischemia differentially regulates LKB1 and an alternate 5′-AMP-activated protein kinase kinase. *J. Biol. Chem.* 280 183–190. 10.1074/jbc.M411810200 15507450

[B6] Amdur R. L. Mukherjee M. Go A. Barrows I. R. Ramezani A. Shoji J. (2016). Interleukin-6 is a risk factor for atrial fibrillation in chronic kidney disease: findings from the CRIC study. *PLoS One* 11:e0148189. 10.1371/journal.pone.0148189 26840403 PMC4739587

[B7] Ancey C. Corbi P. Froger J. Delwail A. Wijdenes J. Gascan H. (2002). Secretion of IL-6. IL-11 and LIF by human cardiomyocytes in primary culture. *Cytokine* 18 199–205. 10.1006/cyto.2002.1033 12126642

[B8] Anderson R. D. Kumar S. Kalman J. M. Sanders P. Sacher F. Hocini M. (2019). Catheter ablation of ventricular fibrillation. *Heart Lung Circ.* 28 110–122. 10.1016/j.hlc.2018.09.005 30301669

[B9] Anstee Q. M. Mantovani A. Tilg H. Targher G. (2018). Risk of cardiomyopathy and cardiac arrhythmias in patients with nonalcoholic fatty liver disease. *Nat. Rev. Gastroenterol. Hepatol.* 15 425–439. 10.1038/s41575-018-0010-0 29713021

[B10] Anto Michel N. Colberg C. Buscher K. Sommer B. Pramod A. B. Ehinger E. (2018). Inflammatory pathways regulated by tumor necrosis receptor-associated factor 1 protect from metabolic consequences in diet-induced obesity. *Circ. Res.* 122 693–700. 10.1161/CIRCRESAHA.117.312055 29358227 PMC5834385

[B11] Anumonwo J. M. B. Herron T. (2018). Fatty infiltration of the myocardium and arrhythmogenesis: potential cellular and molecular mechanisms. *Front. Physiol.* 9:2. 10.3389/fphys.2018.00002 29403390 PMC5786512

[B12] Arbour N. C. Lorenz E. Schutte B. C. Zabner J. Kline J. N. Jones M. (2000). TLR4 mutations are associated with endotoxin hyporesponsiveness in humans. *Nat. Genet.* 25 187–191. 10.1038/76048 10835634

[B13] Aromolaran A. S. Boutjdir M. (2017). Cardiac ion channel regulation in obesity and the metabolic syndrome: relevance to long qt syndrome and atrial fibrillation. *Front. Physiol.* 8:431. 10.3389/fphys.2017.00431 28680407 PMC5479057

[B14] Aromolaran A. S. Colecraft H. M. Boutjdir M. (2016). High-fat diet-dependent modulation of the delayed rectifier K(+) current in adult guinea pig atrial myocytes. *Biochem. Biophys. Res. Commun.* 474 554–559. 10.1016/j.bbrc.2016.04.113 27130822

[B15] Aromolaran A. S. Subramanyam P. Chang D. D. Kobertz W. R. Colecraft H. M. (2014). LQT1 mutations in KCNQ1 C-terminus assembly domain suppress IKs using different mechanisms. *Cardiovasc. Res.* 104 501–511. 10.1093/cvr/cvu231 25344363 PMC4296111

[B16] Ashrafi R. Modi P. Oo A. Y. Pullan D. M. Jian K. Zhang H. (2017). Arrhythmogenic gene remodelling in elderly patients with type 2 diabetes with aortic stenosis and normal left ventricular ejection fraction. *Exp. Physiol.* 102 1424–1434. 10.1113/EP086412 28804970

[B17] Aulin J. Siegbahn A. Hijazi Z. Ezekowitz M. D. Andersson U. Connolly S. J. (2015). Interleukin-6 and C-reactive protein and risk for death and cardiovascular events in patients with atrial fibrillation. *Am. Heart. J* 170 1151–1160. 10.1016/j.ahj.2015.09.018 26678637

[B18] Avlas O. Bragg A. Fuks A. Nicholson J. D. Farkash A. Porat E. (2015). TLR4 expression is associated with left ventricular dysfunction in patients undergoing coronary artery bypass surgery. *PLoS One* 10:e0120175. 10.1371/journal.pone.0120175 26030867 PMC4451004

[B19] Avlas O. Fallach R. Shainberg A. Porat E. Hochhauser E. (2011). Toll-like receptor 4 stimulation initiates an inflammatory response that decreases cardiomyocyte contractility. *Antioxid. Redox. Signal.* 15 1895–1909. 10.1089/ars.2010.3728 21126202

[B20] Bashir S. Sharma Y. Elahi A. Khan F. (2016). Amelioration of obesity-associated inflammation and insulin resistance in c57bl/6 mice via macrophage polarization by fish oil supplementation. *J. Nutr. Biochem.* 33 82–90. 10.1016/j.jnutbio.2016.02.011 27260471

[B21] Beitland S. Platou E. S. Sunde K. (2014). Drug-induced long QT syndrome and fatal arrhythmias in the intensive care unit. *Acta Anaesthesiol. Scand.* 58 266–272. 10.1111/aas.12257 24397608

[B22] Bers D. M. Despa S. (2009). Na+ transport in cardiac myocytes; Implications for excitation-contraction coupling. *IUBMB Life* 61 215–221. 10.1002/iub.163 19243007 PMC2669704

[B23] Billman G. E. Kang J. X. Leaf A. (1999). Prevention of sudden cardiac death by dietary pure omega-3 polyunsaturated fatty acids in dogs. *Circulation* 99 2452–2457. 10.1161/01.CIR.99.18.2452 10318669

[B24] Bjorkbacka H. Kunjathoor V. V. Moore K. J. Koehn S. Ordija C. M. Lee M. A. (2004). Reduced atherosclerosis in MyD88-null mice links elevated serum cholesterol levels to activation of innate immunity signaling pathways. *Nat. Med.* 10 416–421. 10.1038/nm1008 15034566

[B25] Blondeau N. Petrault O. Manta S. Giordanengo V. Gounon P. Bordet R. (2007). Polyunsaturated fatty acids are cerebral vasodilators via the TREK-1 potassium channel. *Circ. Res.* 101 176–184. 10.1161/CIRCRESAHA.107.154443 17556656

[B26] Bogdanov K. Y. Spurgeon H. A. Vinogradova T. M. Lakatta E. G. (1998). Modulation of the transient outward current in adult rat ventricular myocytes by polyunsaturated fatty acids. *Am. J. Physiol.* 274 H571–H579. 10.1152/ajpheart.1998.274.2.H571 9486261

[B27] Boos C. J. Lip G. Y. Jilma B. (2007). Endotoxemia, inflammation, and atrial fibrillation. *Am. J. Cardiol.* 100 986–988. 10.1016/j.amjcard.2007.04.039 17826383

[B28] Borjesson S. I. Elinder F. (2011). An electrostatic potassium channel opener targeting the final voltage sensor transition. *J. Gen. Physiol.* 137 563–577. 10.1085/jgp.201110599 21624947 PMC3105513

[B29] Borjesson S. I. Parkkari T. Hammarstrom S. Elinder F. (2010). Electrostatic tuning of cellular excitability. *Biophys. J.* 98 396–403. 10.1016/j.bpj.2009.10.026 20141752 PMC2814211

[B30] Bosisio D. Polentarutti N. Sironi M. Bernasconi S. Miyake K. Webb G. R. (2002). Stimulation of toll-like receptor 4 expression in human mononuclear phagocytes by interferon-gamma: a molecular basis for priming and synergism with bacterial lipopolysaccharide. *Blood* 99 3427–3431. 10.1182/blood.V99.9.3427 11964313

[B31] Bradley R. L. Fisher F. F. Maratos-Flier E. (2008). Dietary fatty acids differentially regulate production of TNF-alpha and IL-10 by murine 3T3-L1 adipocytes. *Obesity* 16 938–944. 10.1038/oby.2008.39 18356844 PMC4862864

[B32] Bunn R. C. Cockrell G. E. Ou Y. Thrailkill K. M. Lumpkin C. K. Jr. Fowlkes J. L. (2010). Palmitate and insulin synergistically induce IL-6 expression in human monocytes. *Cardiovasc. Diabetol.* 9:73. 10.1186/1475-2840-9-73 21054880 PMC2988002

[B33] Castelli M. Panerai A. Sacerdote P. Franchi S. (2015). Measurement of macrophage toll-like receptor 4 expression after morphine treatment. *Methods Mol. Biol.* 1230 263–271. 10.1007/978-1-4939-1708-2_22 25293333

[B34] Chen X. Bing Z. He J. Jiang L. Luo X. Su Y. (2009). Downregulation of peroxisome proliferator-activated receptor-gamma expression in hypertensive atrial fibrillation. *Clin. Cardiol.* 32 337–345. 10.1002/clc.20566 19569080 PMC6653386

[B35] Cheng E. P. Yuan C. Navedo M. F. Dixon R. E. Nieves-Cintron M. Scott J. D. (2011). Restoration of normal L-type Ca2+ channel function during Timothy syndrome by ablation of an anchoring protein. *Circ. Res.* 109 255–261. 10.1161/CIRCRESAHA.111.248252 21700933 PMC3151468

[B36] Cheng T. Wang X. F. Hou Y. T. Zhang L. (2012). Correlation between atrial fibrillation, serum amyloid protein A and other inflammatory cytokines. *Mol. Med. Rep.* 6 581–584. 10.3892/mmr.2012.934 22684635

[B37] Chimenti C. Verardo R. Scopelliti F. Grande C. Petrosillo N. Piselli P. (2017). Myocardial expression of Toll-like receptor 4 predicts the response to immunosuppressive therapy in patients with virus-negative chronic inflammatory cardiomyopathy. *Eur. J. Heart Fail.* 19 915–925. 10.1002/ejhf.796 28370906

[B38] Chiu H. C. Kovacs A. Blanton R. M. Han X. Courtois M. Weinheimer C. J. (2005). Transgenic expression of fatty acid transport protein 1 in the heart causes lipotoxic cardiomyopathy. *Circ. Res.* 96 225–233. 10.1161/01.RES.0000154079.20681.B9 15618539

[B39] Choudhary S. Kalita M. Fang L. Patel K. V. Tian B. Zhao Y. (2013). Inducible tumor necrosis factor (TNF) receptor-associated factor-1 expression couples the canonical to the non-canonical NF-kappaB pathway in TNF stimulation. *J. Biol. Chem.* 288 14612–14623. 10.1074/jbc.M113.464081 23543740 PMC3656313

[B40] Conway D. S. Buggins P. Hughes E. Lip G. Y. (2004). Prognostic significance of raised plasma levels of interleukin-6 and C-reactive protein in atrial fibrillation. *Am. Heart J.* 148 462–466. 10.1016/j.ahj.2004.01.026 15389233

[B41] Cruz-Teno C. Perez-Martinez P. Delgado-Lista J. Yubero-Serrano E. M. Garcia-Rios A. Marin C. (2012). Dietary fat modifies the postprandial inflammatory state in subjects with metabolic syndrome: the LIPGENE study. *Mol. Nutr. Food Res.* 56 854–865. 10.1002/mnfr.201200096 22707261

[B42] Curtiss L. K. Tobias P. S. (2009). Emerging role of Toll-like receptors in atherosclerosis. *J. Lipid Res.* 50(Suppl.), S340–S345. 10.1194/jlr.R800056-JLR200 18980945 PMC2674724

[B43] Dan G. A. Dobrev D. (2018). Antiarrhythmic drugs for atrial fibrillation: imminent impulses are emerging. *Int. J. Cardiol. Heart Vasc.* 21 11–15. 10.1016/j.ijcha.2018.08.005 30225340 PMC6138883

[B44] Dange R. B. Agarwal D. Masson G. S. Vila J. Wilson B. Nair A. (2014). Central blockade of TLR4 improves cardiac function and attenuates myocardial inflammation in angiotensin II-induced hypertension. *Cardiovasc. Res.* 103 17–27. 10.1093/cvr/cvu067 24667851

[B45] Das R. Burke T. Van Wagoner D. R. Plow E. F. (2009). L-type calcium channel blockers exert an antiinflammatory effect by suppressing expression of plasminogen receptors on macrophages. *Circ. Res.* 105 167–175. 10.1161/CIRCRESAHA.109.200311 19520970 PMC2745969

[B46] Das S. Reddy M. A. Senapati P. Stapleton K. Lanting L. Wang M. (2018). Diabetes mellitus-induced long noncoding RNA Dnm3os regulates macrophage functions and inflammation via nuclear mechanisms. *Arterioscler. Thromb. Vasc. Biol.* 38 1806–1820. 10.1161/ATVBAHA.117.310663 29930005 PMC6202204

[B47] Davis J. E. Gabler N. K. Walker-Daniels J. Spurlock M. E. (2008). Tlr-4 deficiency selectively protects against obesity induced by diets high in saturated fat. *Obesity* 16 1248–1255. 10.1038/oby.2008.210 18421279

[B48] De Coster T. Claus P. Kazbanov I. V. Haemers P. Willems R. Sipido K. R. (2018). Arrhythmogenicity of fibro-fatty infiltrations. *Sci. Rep.* 8:2050. 10.1038/s41598-018-20450-w 29391548 PMC5795000

[B49] Devaraj S. Dasu M. R. Rockwood J. Winter W. Griffen S. C. Jialal I. (2008). Increased toll-like receptor (TLR) 2 and TLR4 expression in monocytes from patients with type 1 diabetes: further evidence of a proinflammatory state. *J. Clin. Endocrinol. Metab.* 93 578–583. 10.1210/jc.2007-2185 18029454 PMC2243229

[B50] Ding S. Jiang J. Wang Z. Zhang G. Yin J. Wang X. (2018). Resveratrol reduces the inflammatory response in adipose tissue and improves adipose insulin signaling in high-fat diet-fed mice. *PeerJ* 6:e5173. 10.7717/peerj.5173 29967759 PMC6027658

[B51] Ding Y. Subramanian S. Montes V. N. Goodspeed L. Wang S. Han C. (2012). Toll-like receptor 4 deficiency decreases atherosclerosis but does not protect against inflammation in obese low-density lipoprotein receptor-deficient mice. *Arterioscler. Thromb. Vasc. Biol.* 32 1596–1604. 10.1161/ATVBAHA.112.249847 22580897 PMC3748807

[B52] Dong B. Qi D. Yang L. Huang Y. Xiao X. Tai N. (2012). TLR4 regulates cardiac lipid accumulation and diabetic heart disease in the nonobese diabetic mouse model of type 1 diabetes. *Am. J. Physiol. Heart Circ. Physiol.* 303 H732–H742. 10.1152/ajpheart.00948.2011 22842069 PMC3468457

[B53] Dong H. Ji Z. Liu M. Wang Y. Bai X. Wang T. (2013). Functional expression of ERG1 potassium channels in rat alveolar macrophages. *J. Mol. Histol.* 44 117–124. 10.1007/s10735-012-9458-3 23138560

[B54] Dosch M. Gerber J. Jebbawi F. Beldi G. (2018). Mechanisms of ATP Release by Inflammatory Cells. *Int. J. Mol. Sci.* 19:E1222. 10.3390/ijms19041222 29669994 PMC5979498

[B55] Drucker C. Gewiese J. Malchow S. Scheller J. Rose-John S. (2010). Impact of interleukin-6 classic- and trans-signaling on liver damage and regeneration. *J. Autoimmun.* 34 29–37. 10.1016/j.jaut.2009.08.003 19717281

[B56] Eckel R. H. Jakicic J. M. Ard J. D. De Jesus J. M. Houston Miller N. Hubbard V. S. (2014). 2013 AHA/ACC guideline on lifestyle management to reduce cardiovascular risk: a report of the American College of Cardiology/American Heart Association Task Force on Practice Guidelines. *J. Am. Coll. Cardiol.* 63 2960–2984. 10.1016/j.jacc.2013.11.003 24239922

[B57] Elinder F. Liin S. I. (2017). Actions and mechanisms of polyunsaturated fatty acids on voltage-gated ion channels. *Front. Physiol.* 8:43. 10.3389/fphys.2017.00043 28220076 PMC5292575

[B58] El-Sherif N. Turitto G. Boutjdir M. (2017). Congenital Long QT syndrome and torsade de pointes. *Ann. Noninvasive Electrocardiol.* 22:e12481. 10.1111/anec.12481 28670758 PMC6931590

[B59] Eranti A. Kerola T. Aro A. L. Tikkanen J. T. Rissanen H. A. Anttonen O. (2016). Diabetes, glucose tolerance, and the risk of sudden cardiac death. *BMC Cardiovasc. Disord.* 16:51. 10.1186/s12872-016-0231-5 26905276 PMC4765126

[B60] Ertunc M. E. Hotamisligil G. S. (2016). Lipid signaling and lipotoxicity in metaflammation: indications for metabolic disease pathogenesis and treatment. *J. Lipid Res.* 57 2099–2114. 10.1194/jlr.R066514 27330055 PMC5321214

[B61] Fernandez-Velasco M. Ruiz-Hurtado G. Hurtado O. Moro M. A. Delgado C. (2007). TNF-alpha downregulates transient outward potassium current in rat ventricular myocytes through iNOS overexpression and oxidant species generation. *Am. J. Physiol. Heart Circ. Physiol.* 293 H238–H245. 10.1152/ajpheart.01122.2006 17337591

[B62] Ferrucci L. Fabbri E. (2018). Inflammageing: chronic inflammation in ageing, cardiovascular disease, and frailty. *Nat. Rev. Cardiol.* 15 505–522. 10.1038/s41569-018-0064-2 30065258 PMC6146930

[B63] Finkel M. S. Hoffman R. A. Shen L. Oddis C. V. Simmons R. L. Hattler B. G. (1993). Interleukin-6 (Il-6) as a mediator of stunned myocardium. *Am. J. Cardiol.* 71 1231–1232. 10.1016/0002-9149(93)90654-U 8480654

[B64] Finkel M. S. Oddis C. V. Jacob T. D. Watkins S. C. Hattler B. G. Simmons R. L. (1992). Negative inotropic effects of cytokines on the heart mediated by nitric oxide. *Science* 257 387–389. 10.1126/science.1631560 1631560

[B65] Flagg T. P. Cazorla O. Remedi M. S. Haim T. E. Tones M. A. Bahinski A. (2009). Ca2+-independent alterations in diastolic sarcomere length and relaxation kinetics in a mouse model of lipotoxic diabetic cardiomyopathy. *Circ. Res.* 104 95–103. 10.1161/CIRCRESAHA.108.186809 19023131

[B66] Fontes J. A. Rose N. R. Cihakova D. (2015). The varying faces of IL-6: from cardiac protection to cardiac failure. *Cytokine* 74 62–68. 10.1016/j.cyto.2014.12.024 25649043 PMC4677779

[B67] Ford J. Milnes J. Wettwer E. Christ T. Rogers M. Sutton K. (2013). Human electrophysiological and pharmacological properties of XEN-D0101: a novel atrial-selective Kv1.5/IKur inhibitor. *J. Cardiovasc. Pharmacol.* 61 408–415. 10.1097/FJC.0b013e31828780eb 23364608

[B68] Frantz S. Kobzik L. Kim Y. D. Fukazawa R. Medzhitov R. Lee R. T. (1999). Toll4 (TLR4) expression in cardiac myocytes in normal and failing myocardium. *J. Clin. Invest.* 104 271–280. 10.1172/JCI6709 10430608 PMC408420

[B69] Fredj S. Lindegger N. Sampson K. J. Carmeliet P. Kass R. S. (2006). Altered Na+ channels promote pause-induced spontaneous diastolic activity in long QT syndrome type 3 myocytes. *Circ. Res.* 99 1225–1232. 10.1161/01.RES.0000251305.25604.b0 17082480 PMC4454351

[B70] Frydrych L. M. Bian G. O’lone D. E. Ward P. A. Delano M. J. (2018). Obesity and type 2 diabetes mellitus drive immune dysfunction, infection development, and sepsis mortality. *J. Leukoc. Biol.* 104 525–534. 10.1002/JLB.5VMR0118-021RR 30066958

[B71] Fuentes-Antras J. Ioan A. M. Tunon J. Egido J. Lorenzo O. (2014). Activation of toll-like receptors and inflammasome complexes in the diabetic cardiomyopathy-associated inflammation. *Int. J. Endocrinol.* 2014:847827. 10.1155/2014/847827 24744784 PMC3972909

[B72] Fujiki A. Sakamoto T. Nishida K. Mizumaki K. Inoue H. (2007). Relation of interleukin-6 and C-reactive protein levels to sinus maintenance after pharmacological cardioversion in persistent atrial fibrillation. *J. Cardiovasc. Pharmacol.* 50 264–266. 10.1097/FJC.0b013e318074f952 17878753

[B73] Gao J. M. Meng X. W. Zhang J. Chen W. R. Xia F. Peng K. (2017). Dexmedetomidine protects cardiomyocytes against hypoxia/reoxygenation injury by suppressing TLR4-MyD88-NF-kappaB Signaling. *Biomed. Res. Int.* 2017:1674613. 10.1155/2017/1674613 29359143 PMC5735617

[B74] Garg P. Garg V. Shrestha R. Sanguinetti M. C. Kamp T. J. Wu J. C. (2018). Human induced pluripotent stem cell-derived cardiomyocytes as models for cardiac channelopathies: a primer for non-electrophysiologists. *Circ. Res.* 123 224–243. 10.1161/CIRCRESAHA.118.311209 29976690 PMC6136439

[B75] Gedikli O. Dogan A. Altuntas I. Altinbas A. Ozaydin M. Akturk O. (2007). Inflammatory markers according to types of atrial fibrillation. *Int. J. Cardiol.* 120 193–197. 10.1016/j.ijcard.2006.09.015 17240468

[B76] Ghanim H. Green K. Abuaysheh S. Patel R. Batra M. Chaudhuri A. (2017). Ezetimibe and simvastatin combination inhibits and reverses the pro-inflammatory and pro-atherogenic effects of cream in obese patients. *Atherosclerosis* 263 278–286. 10.1016/j.atherosclerosis.2017.06.010 28711708

[B77] Gicquel T. Le Dare B. Boichot E. Lagente V. (2017). Purinergic receptors: new targets for the treatment of gout and fibrosis. *Fundam. Clin. Pharmacol.* 31 136–146. 10.1111/fcp.12256 27885718

[B78] Glenn D. J. Wang F. Nishimoto M. Cruz M. C. Uchida Y. Holleran W. M. (2011). A murine model of isolated cardiac steatosis leads to cardiomyopathy. *Hypertension* 57 216–222. 10.1161/HYPERTENSIONAHA.110.160655 21220706 PMC3322545

[B79] Goonasekara C. L. Balse E. Hatem S. Steele D. F. Fedida D. (2010). Cholesterol and cardiac arrhythmias. *Expert Rev. Cardiovasc. Ther.* 8 965–979. 10.1586/erc.10.79 20602558

[B80] Grandi E. Sanguinetti M. C. Bartos D. C. Bers D. M. Chen-Izu Y. Chiamvimonvat N. (2017). Potassium channels in the heart: structure, function and regulation. *J. Physiol.* 595 2209–2228. 10.1113/JP272864 27861921 PMC5374109

[B81] Grandy S. A. Fiset C. (2009). Ventricular K+ currents are reduced in mice with elevated levels of serum TNFalpha. *J. Mol. Cell Cardiol.* 47 238–246. 10.1016/j.yjmcc.2009.02.025 19281815

[B82] Grouthier V. Lebrun-Vignes B. Glazer A. M. Touraine P. Funck-Brentano C. Pariente A. (2018). Increased long QT and torsade de pointes reporting on tamoxifen compared with aromatase inhibitors. *Heart* 104 1859–1863. 10.1136/heartjnl-2017-312934 29720397 PMC8022857

[B83] Gupta S. Sen S. (2005). Role of the NF-kappaB signaling cascade and NF-kappaB-targeted genes in failing human hearts. *J. Mol. Med.* 83 993–1004. 10.1007/s00109-005-0691-z 16133425

[B84] Guzzardi M. A. Iozzo P. (2011). Fatty heart, cardiac damage, and inflammation. *Rev. Diabet. Stud.* 8 403–417. 10.1900/RDS.2011.8.403 22262077 PMC3280674

[B85] Haffar T. Berube-Simard F. A. Bousette N. (2015). Cardiomyocyte lipotoxicity is mediated by Il-6 and causes down-regulation of PPARs. *Biochem. Biophys. Res. Commun.* 459 54–59. 10.1016/j.bbrc.2015.02.062 25712520

[B86] Hagiwara Y. Miyoshi S. Fukuda K. Nishiyama N. Ikegami Y. Tanimoto K. (2007). SHP2-mediated signaling cascade through gp130 is essential for Lif-dependent I CaL, [Ca2+]i transient, and APD increase in cardiomyocytes. *J. Mol. Cell Cardiol.* 43 710–716. 10.1016/j.yjmcc.2007.09.004 17961593

[B87] Haim T. E. Wang W. Flagg T. P. Tones M. A. Bahinski A. Numann R. E. (2010). Palmitate attenuates myocardial contractility through augmentation of repolarizing Kv currents. *J. Mol. Cell Cardiol.* 48 395–405. 10.1016/j.yjmcc.2009.10.004 19857498 PMC2813364

[B88] Hatada K. Washizuka T. Horie M. Watanabe H. Yamashita F. Chinushi M. (2006). Tumor necrosis factor-alpha inhibits the cardiac delayed rectifier K current via the asphingomyelin pathway. *Biochem. Biophys. Res. Commun.* 344 189–193. 10.1016/j.bbrc.2006.03.115 16615994

[B89] He Y. Ma N. Tang M. Jiang Z. L. Liu H. Mei J. (2017). The differentiation of beige adipocyte in pericardial and epicardial adipose tissues induces atrial fibrillation development. *Eur. Rev. Med. Pharmacol. Sci.* 21 4398–4405. 29077154

[B90] Heijman J. Guichard J. B. Dobrev D. Nattel S. (2018). Translational challenges in atrial fibrillation. *Circ. Res.* 122 752–773. 10.1161/CIRCRESAHA.117.311081 29496798

[B91] Heinrich P. C. Behrmann I. Haan S. Hermanns H. M. Muller-Newen G. Schaper F. (2003). Principles of interleukin (IL)-6-type cytokine signalling and its regulation. *Biochem. J.* 374 1–20. 10.1042/bj20030407 12773095 PMC1223585

[B92] Henao-Mejia J. Elinav E. Jin C. Hao L. Mehal W. Z. Strowig T. (2012). Inflammasome-mediated dysbiosis regulates progression of NAFLD and obesity. *Nature* 482 179–185. 10.1038/nature10809 22297845 PMC3276682

[B93] Henningsen K. M. Therkelsen S. K. Bruunsgaard H. Krabbe K. S. Pedersen B. K. Svendsen J. H. (2009). Prognostic impact of hs-CRP and IL-6 in patients with persistent atrial fibrillation treated with electrical cardioversion. *Scand. J. Clin. Lab. Invest.* 69 425–432. 10.1080/00365510802676848 19204850

[B94] Hirano K. Ikeda Y. Zaima N. Sakata Y. Matsumiya G. (2008). Triglyceride deposit cardiomyovasculopathy. *N. Engl. J. Med.* 359 2396–2398. 10.1056/NEJMc0805305 19038890

[B95] Hoy A. J. Bruce C. R. Turpin S. M. Morris A. J. Febbraio M. A. Watt M. J. (2011). Adipose triglyceride lipase-null mice are resistant to high-fat diet-induced insulin resistance despite reduced energy expenditure and ectopic lipid accumulation. *Endocrinology* 152 48–58. 10.1210/en.2010-0661 21106876

[B96] Hsiao P. Y. Tien H. C. Lo C. P. Juang J. M. Wang Y. H. Sung R. J. (2013). Gene mutations in cardiac arrhythmias: a review of recent evidence in ion channelopathies. *Appl. Clin. Genet.* 6 1–13. 10.2147/TACG.S29676 23837003 PMC3699290

[B97] Huang S. Rutkowsky J. M. Snodgrass R. G. Ono-Moore K. D. Schneider D. A. Newman J. W. (2012). Saturated fatty acids activate TLR-mediated proinflammatory signaling pathways. *J. Lipid Res.* 53 2002–2013. 10.1194/jlr.D029546 22766885 PMC3413240

[B98] Huang T. H. Wang P. W. Yang S. C. Chou W. L. Fang J. Y. (2018). Cosmetic and therapeutic applications of fish oil’s fatty acids on the skin. *Mar. Drugs* 16:E256. 10.3390/md16080256 30061538 PMC6117694

[B99] Huh H. D. Ra E. A. Lee T. A. Kang S. Park A. Lee E. (2016). STRAP Acts as a scaffolding protein in controlling the TLR2/4 signaling pathway. *Sci. Rep.* 6:38849. 10.1038/srep38849 27934954 PMC5146969

[B100] Hulsmans M. Sam F. Nahrendorf M. (2016). Monocyte and macrophage contributions to cardiac remodeling. *J. Mol. Cell Cardiol.* 93 149–155. 10.1016/j.yjmcc.2015.11.015 26593722 PMC4846552

[B101] Hwang D. H. Kim J. A. Lee J. Y. (2016). Mechanisms for the activation of Toll-like receptor 2/4 by saturated fatty acids and inhibition by docosahexaenoic acid. *Eur. J. Pharmacol.* 785 24–35. 10.1016/j.ejphar.2016.04.024 27085899 PMC5815395

[B102] Iacobellis G. Bianco A. C. (2011). Epicardial adipose tissue: emerging physiological, pathophysiological and clinical features. *Trends Endocrinol. Metab.* 22 450–457. 10.1016/j.tem.2011.07.003 21852149 PMC4978122

[B103] Ihle J. N. (1995). Cytokine receptor signalling. *Nature* 377 591–594. 10.1038/377591a0 7566171

[B104] Jeevaratnam K. Chadda K. R. Huang C. L. Camm A. J. (2018). Cardiac potassium channels: physiological insights for targeted therapy. *J. Cardiovasc. Pharmacol. Ther.* 23 119–129. 10.1177/1074248417729880 28946759 PMC5808825

[B105] Jiang H. Qu P. (2017). Effects of Ginkgo biloba leaf extract on local renin-angiotensin system through TLR4/NF-kappaB pathway in cardiac myocyte. *Exp. Ther. Med.* 14 5857–5862. 10.3892/etm.2017.5313 29285133 PMC5740758

[B106] Jiang H. Qu P. Wang J. W. Li G. H. Wang H. Y. (2018). Effect of NF-kappaB inhibitor on Toll-like receptor 4 expression in left ventricular myocardium in two-kidney-one-clip hypertensive rats. *Eur. Rev. Med. Pharmacol. Sci.* 22 3224–3233. 10.26355/eurrev_201805_15084 29863269

[B107] Jin J. Lu Z. Li Y. Ru J. H. Lopes-Virella M. F. Huang Y. (2018). LPS and palmitate synergistically stimulate sphingosine kinase 1 and increase sphingosine 1 phosphate in RAW264.7 macrophages. *J. Leukoc. Biol.* 104 843–853. 10.1002/JLB.3A0517-188RRR 29882996 PMC6162112

[B108] Joshi N. Caputo G. M. Weitekamp M. R. Karchmer A. W. (1999). Infections in patients with diabetes mellitus. *N. Engl. J. Med.* 341 1906–1912. 10.1056/NEJM199912163412507 10601511

[B109] Kan X. H. Gao H. Q. Ma Z. Y. Liu L. Ling M. Y. Wang Y. Y. (2016). Kv1.3 potassium channel mediates macrophage migration in atherosclerosis by regulating ERK activity. *Arch. Biochem. Biophys.* 591 150–156. 10.1016/j.abb.2015.12.013 26748289

[B110] Kawada H. Niwano S. Niwano H. Yumoto Y. Wakisaka Y. Yuge M. (2006). Tumor necrosis factor-alpha downregulates the voltage gated outward K+ current in cultured neonatal rat cardiomyocytes: a possible cause of electrical remodeling in diseased hearts. *Circ. J.* 70 605–609. 10.1253/circj.70.605 16636498

[B111] Kawamura N. Kubota T. Kawano S. Monden Y. Feldman A. M. Tsutsui H. (2005). Blockade of NF-kappaB improves cardiac function and survival without affecting inflammation in TNF-alpha-induced cardiomyopathy. *Cardiovasc. Res.* 66 520–529. 10.1016/j.cardiores.2005.02.007 15914117

[B112] Kiechl S. Lorenz E. Reindl M. Wiedermann C. J. Oberhollenzer F. Bonora E. (2002). Toll-like receptor 4 polymorphisms and atherogenesis. *N. Engl. J. Med.* 347 185–192. 10.1056/NEJMoa012673 12124407

[B113] Kien C. L. Bunn J. Y. Ugrasbul F. (2005). Increasing dietary palmitic acid decreases fat oxidation and daily energy expenditure. *Am. J. Clin. Nutr.* 82 320–326. 10.1093/ajcn/82.2.320 16087974 PMC1314972

[B114] Kim F. Pham M. Luttrell I. Bannerman D. D. Tupper J. Thaler J. (2007). Toll-like receptor-4 mediates vascular inflammation and insulin resistance in diet-induced obesity. *Circ. Res.* 100 1589–1596. 10.1161/CIRCRESAHA.106.142851 17478729

[B115] Kim J. A. Lopes C. M. Moss A. J. Mcnitt S. Barsheshet A. Robinson J. L. (2010). Trigger-specific risk factors and response to therapy in long QT syndrome type 2. *Heart Rhythm* 7 1797–1805. 10.1016/j.hrthm.2010.09.011 20850565 PMC3032939

[B116] Kinugawa K. Takahashi T. Kohmoto O. Yao A. Aoyagi T. Momomura S. (1994). Nitric oxide-mediated effects of interleukin-6 on [Ca2+]i and cell contraction in cultured chick ventricular myocytes. *Circ. Res.* 75 285–295. 10.1161/01.RES.75.2.285 7518362

[B117] Krauss R. M. Eckel R. H. Howard B. Appel L. J. Daniels S. R. Deckelbaum R. J. (2000). AHA dietary guidelines: revision 2000: a statement for healthcare professionals from the nutrition committee of the American Heart Association. *Circulation* 102 2284–2299. 10.1161/01.CIR.102.18.2284 11056107

[B118] Kumar A. Thota V. Dee L. Olson J. Uretz E. Parrillo J. E. (1996). Tumor necrosis factor alpha and interleukin 1beta are responsible for *in vitro* myocardial cell depression induced by human septic shock serum. *J. Exp. Med.* 183 949–958. 10.1084/jem.183.3.949 8642298 PMC2192364

[B119] Lazzerini P. E. Acampa M. Capecchi P. L. Fineschi I. Selvi E. Moscadelli V. (2015a). Antiarrhythmic potential of anticytokine therapy in rheumatoid arthritis: tocilizumab reduces corrected QT interval by controlling systemic inflammation. *Arthritis Care Res.* 67 332–339. 10.1002/acr.22455 25186226

[B120] Lazzerini P. E. Capecchi P. L. Acampa M. Galeazzi M. Laghi-Pasini F. (2014). Arrhythmic risk in rheumatoid arthritis: the driving role of systemic inflammation. *Autoimmun. Rev.* 13 936–944. 10.1016/j.autrev.2014.05.007 24874445

[B121] Lazzerini P. E. Capecchi P. L. Bertolozzi I. Morozzi G. Lorenzini S. Simpatico A. (2016). Marked QTc prolongation and torsades de pointes in patients with chronic inflammatory arthritis. *Front. Cardiovasc. Med.* 3:31. 10.3389/fcvm.2016.00031 27703966 PMC5029147

[B122] Lazzerini P. E. Capecchi P. L. Boutjdir M. Laghi-Pasini F. (2015b). Comment on “absence of an association between anti-Ro antibodies and prolonged QTc interval in systemic sclerosis: a multicenter study of 689 patients”. *Semin. Arthritis Rheum.* 44 e16–e17. 10.1016/j.semarthrit.2014.10.002 25455682

[B123] Lazzerini P. E. Capecchi P. L. Laghi-Pasini F. (2015c). Long QT Syndrome: an emerging role for inflammation and immunity. *Front. Cardiovasc. Med.* 2:26. 10.3389/fcvm.2015.00026 26798623 PMC4712633

[B124] Lazzerini P. E. Capecchi P. L. Laghi-Pasini F. (2017a). Systemic inflammation and arrhythmic risk: lessons from rheumatoid arthritis. *Eur. Heart J.* 38 1717–1727. 10.1093/eurheartj/ehw208 27252448

[B125] Lazzerini P. E. Laghi-Pasini F. Bertolozzi I. Morozzi G. Lorenzini S. Simpatico A. (2017b). Systemic inflammation as a novel QT-prolonging risk factor in patients with torsades de pointes. *Heart* 103 1821–1829. 10.1136/heartjnl-2016-311079 28490617

[B126] Leaf A. (2007). Prevention of sudden cardiac death by n-3 polyunsaturated fatty acids. *J. Cardiovasc. Med.* 8(Suppl. 1), S27–S29. 10.2459/01.JCM.0000289270.98105.b3 17876194

[B127] Leaf A. Xiao Y. F. Kang J. X. Billman G. E. (2003). Prevention of sudden cardiac death by n-3 polyunsaturated fatty acids. *Pharmacol. Ther.* 98 355–377. 10.1016/S0163-7258(03)00039-1 12782244

[B128] Lee A. H. Ledderose C. Li X. Slubowski C. J. Sueyoshi K. Staudenmaier L. (2018). Adenosine triphosphate release is required for toll-like receptor-induced monocyte/macrophage activation, inflammasome signaling, interleukin-1beta production, and the host immune response to infection. *Crit. Care Med.* e1183–e1189. 10.1097/CCM.0000000000003446 30247270 PMC6239954

[B129] Lee J. Y. Sohn K. H. Rhee S. H. Hwang D. (2001). Saturated fatty acids, but not unsaturated fatty acids, induce the expression of cyclooxygenase-2 mediated through Toll-like receptor 4. *J. Biol. Chem.* 276 16683–16689. 10.1074/jbc.M011695200 11278967

[B130] Lee J. Y. Ye J. Gao Z. Youn H. S. Lee W. H. Zhao L. (2003). Reciprocal modulation of Toll-like receptor-4 signaling pathways involving MyD88 and phosphatidylinositol 3-kinase/AKT by saturated and polyunsaturated fatty acids. *J. Biol. Chem.* 278 37041–37051. 10.1074/jbc.M305213200 12865424

[B131] Lee J. Y. Zhao L. Youn H. S. Weatherill A. R. Tapping R. Feng L. (2004). Saturated fatty acid activates but polyunsaturated fatty acid inhibits Toll-like receptor 2 dimerized with Toll-like receptor 6 or 1. *J. Biol. Chem.* 279 16971–16979. 10.1074/jbc.M312990200 14966134

[B132] Lefebvre P. J. Scheen A. J. (1998). The postprandial state and risk of cardiovascular disease. *Diabet. Med.* 15(Suppl. 4), S63–S68. 10.1002/(SICI)1096-9136(1998120)15:4+<S63::AID-DIA737>3.3.CO;2-Z 9868996

[B133] Leftheriotis D. I. Fountoulaki K. T. Flevari P. G. Parissis J. T. Panou F. K. Andreadou I. T. (2009). The predictive value of inflammatory and oxidative markers following the successful cardioversion of persistent lone atrial fibrillation. *Int. J. Cardiol.* 135 361–369. 10.1016/j.ijcard.2008.04.012 18640731

[B134] Lewis A. J. M. Miller J. J. Lau A. Z. Curtis M. K. Rider O. J. Choudhury R. P. (2018). Noninvasive immunometabolic cardiac inflammation imaging using hyperpolarized magnetic resonance. *Circ. Res.* 122 1084–1093. 10.1161/CIRCRESAHA.117.312535 29440071 PMC5908252

[B135] Liang C. F. Liu J. T. Wang Y. Xu A. Vanhoutte P. M. (2013). Toll-like receptor 4 mutation protects obese mice against endothelial dysfunction by decreasing NADPH oxidase isoforms 1 and 4. *Arterioscler. Thromb. Vasc. Biol.* 33 777–784. 10.1161/ATVBAHA.112.301087 23413427

[B136] Liin S. I. Silvera Ejneby M. Barro-Soria R. Skarsfeldt M. A. Larsson J. E. Starck Harlin F. (2015). Polyunsaturated fatty acid analogs act antiarrhythmically on the cardiac IKs channel. *Proc. Natl. Acad. Sci. U.S.A.* 112 5714–5719. 10.1073/pnas.1503488112 25901329 PMC4426425

[B137] Lin Y. T. Verma A. Hodgkinson C. P. (2012). Toll-like receptors and human disease: lessons from single nucleotide polymorphisms. *Curr. Genomics* 13 633–645. 10.2174/138920212803759712 23730203 PMC3492803

[B138] Listenberger L. L. Han X. Lewis S. E. Cases S. Farese R. V. Jr. Ory D. S. (2003). Triglyceride accumulation protects against fatty acid-induced lipotoxicity. *Proc. Natl. Acad. Sci. U.S.A.* 100 3077–3082. 10.1073/pnas.0630588100 12629214 PMC152249

[B139] Liu C. Zhang R. Sun C. Zhang H. Xu C. Liu W. (2015). Resveratrol prevents cadmium activation of Erk1/2 and Jnk pathways from neuronal cell death via protein phosphatases 2A and 5. *J. Neurochem.* 135 466–478. 10.1111/jnc.13233 26146868 PMC9178502

[B140] Liu X. Shi J. Xiao P. (2018). Associations between common ion channel single nucleotide polymorphisms and sudden cardiac death in adults: a MOOSE-compliant meta-analysis. *Medicine* 97:e12428. 10.1097/MD.0000000000012428 30235722 PMC6160092

[B141] Liu X. Ukai T. Yumoto H. Davey M. Goswami S. Gibson F. C. III (2008). Toll-like receptor 2 plays a critical role in the progression of atherosclerosis that is independent of dietary lipids. *Atherosclerosis* 196 146–154. 10.1016/j.atherosclerosis.2007.03.025 17466307 PMC2243224

[B142] London B. Baker L. C. Lee J. S. Shusterman V. Choi B. R. Kubota T. (2003). Calcium-dependent arrhythmias in transgenic mice with heart failure. *Am. J. Physiol. Heart Circ. Physiol.* 284 H431–H441. 10.1152/ajpheart.00431.2002 12388316

[B143] Lopez-Pascual A. Lorente-Cebrian S. Moreno-Aliaga M. J. Martinez J. A. Gonzalez-Muniesa P. (2018). Inflammation stimulates hypoxia-inducible factor-1alpha regulatory activity in 3T3-L1 adipocytes with conditioned medium from lipopolysaccharide-activated RAW 264.7 macrophages. *J. Cell Physiol.* 234 550–560. 10.1002/jcp.26763 30071127

[B144] Lu L. Sirish P. Zhang Z. Woltz R. L. Li N. Timofeyev V. (2015). Regulation of gene transcription by voltage-gated L-type calcium channel. Cav1.3. *J. Biol. Chem.* 290 4663–4676. 10.1074/jbc.M114.586883 25538241 PMC4335206

[B145] Lundman P. Boquist S. Samnegard A. Bennermo M. Held C. Ericsson C. G. (2007). A high-fat meal is accompanied by increased plasma interleukin-6 concentrations. *Nutr. Metab. Cardiovasc. Dis.* 17 195–202. 10.1016/j.numecd.2005.11.009 17367705

[B146] Lyons C. L. Kennedy E. B. Roche H. M. (2016). Metabolic inflammation-differential modulation by dietary constituents. *Nutrients* 8:E247. 10.3390/nu8050247 27128935 PMC4882660

[B147] Maass D. L. White J. Horton J. W. (2002). IL-1beta and IL-6 act synergistically with TNF-alpha to alter cardiac contractile function after burn trauma. *Shock* 18 360–366. 10.1097/00024382-200210000-00012 12392281

[B148] Madan M. Amar S. (2008). Toll-like receptor-2 mediates diet and/or pathogen associated atherosclerosis: proteomic findings. *PLoS One* 3:e3204. 10.1371/journal.pone.0003204 18787704 PMC2527517

[B149] Mancarella S. Yue Y. Karnabi E. Qu Y. El-Sherif N. Boutjdir M. (2008). Impaired Ca2+ homeostasis is associated with atrial fibrillation in the alpha1D L-type Ca2+ channel KO mouse. *Am. J. Physiol. Heart Circ. Physiol.* 295 H2017–H2024. 10.1152/ajpheart.00537.2008 18790836 PMC2614575

[B150] Marcus G. M. Smith L. M. Ordovas K. Scheinman M. M. Kim A. M. Badhwar N. (2010). Intracardiac and extracardiac markers of inflammation during atrial fibrillation. *Heart Rhythm* 7 149–154. 10.1016/j.hrthm.2009.10.004 20022819 PMC2900773

[B151] Marcus G. M. Whooley M. A. Glidden D. V. Pawlikowska L. Zaroff J. G. Olgin J. E. (2008). Interleukin-6 and atrial fibrillation in patients with coronary artery disease: data from the Heart and Soul Study. *Am. Heart. J.* 155 303–309. 10.1016/j.ahj.2007.09.006 18215601 PMC2247366

[B152] Marin F. Roldan V. Climent V. E. Ibanez A. Garcia A. Marco P. (2004). Plasma von Willebrand factor, soluble thrombomodulin, and fibrin D-dimer concentrations in acute onset non-rheumatic atrial fibrillation. *Heart* 90 1162–1166. 10.1136/hrt.2003.024521 15367514 PMC1768504

[B153] Mezzaroma E. Toldo S. Farkas D. Seropian I. M. Van Tassell B. W. Salloum F. N. (2011). The inflammasome promotes adverse cardiac remodeling following acute myocardial infarction in the mouse. *Proc. Natl. Acad. Sci. U.S.A.* 108 19725–19730. 10.1073/pnas.1108586108 22106299 PMC3241791

[B154] Michelsen K. S. Wong M. H. Shah P. K. Zhang W. Yano J. Doherty T. M. (2004). Lack of Toll-like receptor 4 or myeloid differentiation factor 88 reduces atherosclerosis and alters plaque phenotype in mice deficient in apolipoprotein E. *Proc. Natl. Acad. Sci. U.S.A.* 101 10679–10684. 10.1073/pnas.0403249101 15249654 PMC489994

[B155] Mihara M. Hashizume M. Yoshida H. Suzuki M. Shiina M. (2012). IL-6/IL-6 receptor system and its role in physiological and pathological conditions. *Clin. Sci.* 122 143–159. 10.1042/CS20110340 22029668

[B156] Mirza R. Dipietro L. A. Koh T. J. (2009). Selective and specific macrophage ablation is detrimental to wound healing in mice. *Am. J. Pathol.* 175 2454–2462. 10.2353/ajpath.2009.090248 19850888 PMC2789630

[B157] Mirza R. Koh T. J. (2011). Dysregulation of monocyte/macrophage phenotype in wounds of diabetic mice. *Cytokine* 56 256–264. 10.1016/j.cyto.2011.06.016 21803601

[B158] Moreno C. Macias A. Prieto A. De La Cruz A. Gonzalez T. Valenzuela C. (2012). Effects of n-3 polyunsaturated fatty acids on cardiac ion channels. *Front. Physiol.* 3:245 10.3389/fphys.2012.00245 PMC3429023 22934003

[B159] Moreno C. Prieto P. Macias A. Pimentel-Santillana M. De La Cruz A. Traves P. G. (2013). Modulation of voltage-dependent and inward rectifier potassium channels by 15-epi-lipoxin-A4 in activated murine macrophages: implications in innate immunity. *J. Immunol.* 191 6136–6146. 10.4049/jimmunol.1300235 24249731

[B160] Mullick A. E. Soldau K. Kiosses W. B. Bell T. A. III Tobias P. S. Curtiss L. K. (2008). Increased endothelial expression of Toll-like receptor 2 at sites of disturbed blood flow exacerbates early atherogenic events. *J. Exp. Med.* 205 373–383. 10.1084/jem.20071096 18250194 PMC2271019

[B161] Mullick A. E. Tobias P. S. Curtiss L. K. (2005). Modulation of atherosclerosis in mice by Toll-like receptor 2. *J. Clin. Invest.* 115 3149–3156. 10.1172/JCI25482 16211093 PMC1242192

[B162] Muser D. Santangeli P. Selvanayagam J. B. Nucifora G. (2018). Role of cardiac magnetic resonance imaging in patients with idiopathic ventricular arrhythmias. *Curr. Cardiol. Rev.* 10.2174/1573403X14666180925095923 [Epub ahead of print]. 30251607 PMC6367696

[B163] Naka T. Narazaki M. Hirata M. Matsumoto T. Minamoto S. Aono A. (1997). Structure and function of a new STAT-induced STAT inhibitor. *Nature* 387 924–929. 10.1038/43219 9202127

[B164] Nattel S. Dobrev D. (2017). Controversies about atrial fibrillation mechanisms: aiming for order in chaos and whether it matters. *Circ. Res.* 120 1396–1398. 10.1161/CIRCRESAHA.116.310489 28450363

[B165] O’Connell R. P. Musa H. Gomez M. S. Avula U. M. Herron T. J. Kalifa J. (2015). Free fatty acid effects on the atrial myocardium: membrane ionic currents are remodeled by the disruption of T-tubular architecture. *PLoS One* 10:e0133052. 10.1371/journal.pone.0133052 26274906 PMC4537212

[B166] Oikonomou E. Vogiatzi G. Karlis D. Siasos G. Chrysohoou C. Zografos T. (2018). Effects of omega-3 polyunsaturated fatty acids on fibrosis, endothelial function and myocardial performance, in ischemic heart failure patients. *Clin. Nutr.* 10.1016/j.clnu.2018.04.017 [Epub ahead of print]. 29752009

[B167] Ozcan C. Battaglia E. Young R. Suzuki G. (2015). LKB1 knockout mouse develops spontaneous atrial fibrillation and provides mechanistic insights into human disease process. *J. Am. Heart Assoc.* 4:e001733. 10.1161/JAHA.114.001733 25773299 PMC4392447

[B168] Pan J. Wang W. Wu X. Kong F. Pan J. Lin J. (2018). Inflammatory cytokines in cardiac pacing patients with atrial fibrillation and asymptomatic atrial fibrillation. *Panminerva Med.* 60 86–91. 10.23736/S0031-0808.18.03452-3 29696963

[B169] Panama B. K. Korogyi A. S. Aschar-Sobbi R. Oh Y. Gray C. B. Gang H. (2016). Reductions in the cardiac transient outward k+ current ito caused by chronic beta-adrenergic receptor stimulation are partly rescued by inhibition of nuclear factor kappaB. *J. Biol. Chem.* 291 4156–4165. 10.1074/jbc.M115.694984 26742842 PMC4759190

[B170] Panama B. K. Latour-Villamil D. Farman G. P. Zhao D. Bolz S. S. Kirshenbaum L. A. (2011). Nuclear factor kappaB downregulates the transient outward potassium current I(to,f) through control of KChIP2 expression. *Circ. Res.* 108 537–543. 10.1161/CIRCRESAHA.110.229112 21252158

[B171] Papaioannou A. Michaloudis D. Fraidakis O. Petrou A. Chaniotaki F. Kanoupakis E. (2003). Effects of weight loss on QT interval in morbidly obese patients. *Obes. Surg.* 13 869–873. 10.1381/096089203322618687 14738673

[B172] Park S. A. Lee Y. C. Ma T. Z. Park J. A. Han M. K. Lee H. H. (2006). hKv1.5 channels play a pivotal role in the functions of human alveolar macrophages. *Biochem. Biophys. Res. Commun.* 346 567–571. 10.1016/j.bbrc.2006.05.149 16765315

[B173] Park T. S. Goldberg I. J. (2012). Sphingolipids, lipotoxic cardiomyopathy, and cardiac failure. *Heart Fail Clin.* 8 633–641. 10.1016/j.hfc.2012.06.003 22999245 PMC4548923

[B174] Pennefather P. S. Zhou W. Decoursey T. E. (1998). Idiosyncratic gating of HERG-like K+ channels in microglia. *J. Gen. Physiol.* 111 795–805. 10.1085/jgp.111.6.795 9607937 PMC2217153

[B175] Petkova-Kirova P. S. Gursoy E. Mehdi H. Mctiernan C. F. London B. Salama G. (2006). Electrical remodeling of cardiac myocytes from mice with heart failure due to the overexpression of tumor necrosis factor-alpha. *Am. J. Physiol. Heart Circ. Physiol.* 290 H2098–H2107. 10.1152/ajpheart.00097.2005 16339842

[B176] Pohl D. Benseler S. (2013). Systemic inflammatory and autoimmune disorders. *Handb. Clin. Neurol.* 112 1243–1252. 10.1016/B978-0-444-52910-7.00047-7 23622335

[B177] Poirier P. Giles T. D. Bray G. A. Hong Y. Stern J. S. Pi-Sunyer F. X. (2006). Obesity and cardiovascular disease: pathophysiology, evaluation, and effect of weight loss: an update of the 1997 American Heart Association Scientific Statement on Obesity and Heart Disease from the Obesity Committee of the Council on Nutrition, Physical Activity, and Metabolism. *Circulation* 113 898–918. 10.1161/CIRCULATIONAHA.106.171016 16380542

[B178] Psychari S. N. Apostolou T. S. Sinos L. Hamodraka E. Liakos G. Kremastinos D. T. (2005). Relation of elevated C-reactive protein and interleukin-6 levels to left atrial size and duration of episodes in patients with atrial fibrillation. *Am. J. Cardiol.* 95 764–767. 10.1016/j.amjcard.2004.11.032 15757607

[B179] Puckerin A. Aromolaran K. A. Chang D. D. Zukin R. S. Colecraft H. M. Boutjdir M. (2016). hERG 1a LQT2 C-terminus truncation mutants display hERG 1b-dependent dominant negative mechanisms. *Heart Rhythm* 13 1121–1130. 10.1016/j.hrthm.2016.01.012 26775140

[B180] Pudil R. Vasatova M. Parizek P. Haman L. Horakova L. Palicka V. (2016). Increase of serum interleukin 6 and interferon gamma is associated with the number of impulses in patients with supraventricular arrhythmias treated with radiofrequency catheter ablation. *Biomed. Pap. Med. Fac. Univ. Palacky. Olomouc. Czech. Repub.* 160 106–110. 10.5507/bp.2015.038 26365928

[B181] Purcell N. H. Tang G. Yu C. Mercurio F. Didonato J. A. Lin A. (2001). Activation of NF-kappa B is required for hypertrophic growth of primary rat neonatal ventricular cardiomyocytes. *Proc. Natl. Acad. Sci. U.S.A.* 98 6668–6673. 10.1073/pnas.111155798 11381115 PMC34410

[B182] Qu Y. C. Du Y. M. Wu S. L. Chen Q. X. Wu H. L. Zhou S. F. (2009). Activated nuclear factor-kappaB and increased tumor necrosis factor-alpha in atrial tissue of atrial fibrillation. *Scand. Cardiovasc. J.* 43 292–297. 10.1080/14017430802651803 19169931

[B183] Rahm A. K. Lugenbiel P. Schweizer P. A. Katus H. A. Thomas D. (2018). Role of ion channels in heart failure and channelopathies. *Biophys. Rev.* 10 1097–1106. 10.1007/s12551-018-0442-3 30019205 PMC6082303

[B184] Ralston J. C. Lyons C. L. Kennedy E. B. Kirwan A. M. Roche H. M. (2017). Fatty Acids and NLRP3 inflammasome-mediated inflammation in metabolic tissues. *Annu. Rev. Nutr.* 37 77–102. 10.1146/annurev-nutr-071816-064836 28826373

[B185] Ramos Muniz M. G. Palfreeman M. Setzu N. Sanchez M. A. Saenz Portillo P. Garza K. M. (2018). Obesity exacerbates the cytokine storm elicited by francisella tularensis infection of females and is associated with increased mortality. *Biomed. Res. Int.* 2018:3412732. 10.1155/2018/3412732 30046592 PMC6038682

[B186] Rennison J. H. Van Wagoner D. R. (2009). Impact of dietary fatty acids on cardiac arrhythmogenesis. *Circ. Arrhythm. Electrophysiol.* 2 460–469. 10.1161/CIRCEP.109.880773 19808503 PMC5831114

[B187] Rimm E. B. Appel L. J. Chiuve S. E. Djousse L. Engler M. B. Kris-Etherton P. M. (2018). Seafood long-chain n-3 polyunsaturated fatty acids and cardiovascular disease: a science advisory from the american heart association. *Circulation* 138 e35–e47. 10.1161/CIR.0000000000000574 29773586 PMC6903778

[B188] Rizos I. Tsiodras S. Rigopoulos A. G. Dragomanovits S. Kalogeropoulos A. S. Papathanasiou S. (2007). Interleukin-2 serum levels variations in recent onset atrial fibrillation are related with cardioversion outcome. *Cytokine* 40 157–164. 10.1016/j.cyto.2007.08.013 17923414

[B189] Sager H. B. Hulsmans M. Lavine K. J. Moreira M. B. Heidt T. Courties G. (2016). Proliferation and recruitment contribute to myocardial macrophage expansion in chronic heart failure. *Circ. Res.* 119 853–864. 10.1161/CIRCRESAHA.116.309001 27444755 PMC5378496

[B190] Sakaguchi M. Murata H. Yamamoto K. Ono T. Sakaguchi Y. Motoyama A. (2011). TIRAP, an adaptor protein for TLR2/4, transduces a signal from RAGE phosphorylated upon ligand binding. *PLoS One* 6:e23132. 10.1371/journal.pone.0023132 21829704 PMC3148248

[B191] Sanchez G. Araneda F. Pena J. P. Finkelstein J. P. Riquelme J. A. Montecinos L. (2018). High-fat-diet-induced obesity produces spontaneous ventricular arrhythmias and increases the activity of ryanodine receptors in mice. *Int. J. Mol. Sci.* 19:533. 10.3390/ijms19020533 29439404 PMC5855755

[B192] Sanguinetti M. C. Jurkiewicz N. K. (1990). Two components of cardiac delayed rectifier K+ current. Differential sensitivity to block by class III antiarrhythmic agents. *J. Gen. Physiol.* 96 195–215. 10.1085/jgp.96.1.195 2170562 PMC2228985

[B193] Sanguinetti M. C. Jurkiewicz N. K. (1991). Delayed rectifier outward K+ current is composed of two currents in guinea pig atrial cells. *Am. J. Physiol.* 260 H393–H399. 10.1152/ajpheart.1991.260.2.H393 1899980

[B194] Santos D. G. Resende M. F. Mill J. G. Mansur A. J. Krieger J. E. Pereira A. C. (2010). Nuclear Factor (NF) kappaB polymorphism is associated with heart function in patients with heart failure. *BMC Med. Genet.* 11:89. 10.1186/1471-2350-11-89 20534156 PMC2897791

[B195] Schilling J. D. Machkovech H. M. He L. Sidhu R. Fujiwara H. Weber K. (2013). Palmitate and lipopolysaccharide trigger synergistic ceramide production in primary macrophages. *J. Biol. Chem.* 288 2923–2932. 10.1074/jbc.M112.419978 23250746 PMC3561515

[B196] Schmocker C. Zhang I. W. Kiesler S. Kassner U. Ostermann A. I. Steinhagen-Thiessen E. (2018). Effect of Omega-3 fatty acid supplementation on oxylipins in a routine clinical setting. *Int. J. Mol. Sci.* 19:E180. 10.3390/ijms19010180 29316682 PMC5796129

[B197] Schreiber F. Lynn D. J. Houston A. Peters J. Mwafulirwa G. Finlay B. B. (2011). The human transcriptome during nontyphoid *Salmonella* and HIV coinfection reveals attenuated NFkappaB-mediated inflammation and persistent cell cycle disruption. *J. Infect. Dis.* 204 1237–1245. 10.1093/infdis/jir512 21917897 PMC3173506

[B198] Schunck W. H. Konkel A. Fischer R. Weylandt K. H. (2018). Therapeutic potential of omega-3 fatty acid-derived epoxyeicosanoids in cardiovascular and inflammatory diseases. *Pharmacol. Ther.* 183 177–204. 10.1016/j.pharmthera.2017.10.016 29080699

[B199] Schwartz P. J. Stramba-Badiale M. Crotti L. Pedrazzini M. Besana A. Bosi G. (2009). Prevalence of the congenital long-QT syndrome. *Circulation* 120 1761–1767. 10.1161/CIRCULATIONAHA.109.863209 19841298 PMC2784143

[B200] Senn J. J. (2006). Toll-like receptor-2 is essential for the development of palmitate-induced insulin resistance in myotubes. *J. Biol. Chem.* 281 26865–26875. 10.1074/jbc.M513304200 16798732

[B201] Shao Y. Redfors B. Stahlman M. Tang M. S. Miljanovic A. Mollmann H. (2013). A mouse model reveals an important role for catecholamine-induced lipotoxicity in the pathogenesis of stress-induced cardiomyopathy. *Eur. J. Heart Fail.* 15 9–22. 10.1093/eurjhf/hfs161 23099354

[B202] Shapiro M. D. Fazio S. (2016). From lipids to inflammation: new approaches to reducing atherosclerotic risk. *Circ. Res.* 118 732–749. 10.1161/CIRCRESAHA.115.306471 26892970

[B203] Sharma S. Adrogue J. V. Golfman L. Uray I. Lemm J. Youker K. (2004). Intramyocardial lipid accumulation in the failing human heart resembles the lipotoxic rat heart. *FASEB J.* 18 1692–1700. 10.1096/fj.04-2263com 15522914

[B204] Shen C. Ma W. Ding L. Li S. Dou X. Song Z. (2018). The TLR4-IRE1alpha pathway activation contributes to palmitate-elicited lipotoxicity in hepatocytes. *J. Cell Mol. Med.* 22 3572–3581. 10.1111/jcmm.13636 29673059 PMC6010797

[B205] Shenthar J. Rachaiah J. M. Pillai V. Chakali S. S. Balasubramanian V. Chollenhalli Nanjappa M. (2017). Incidence of drug-induced torsades de pointes with intravenous amiodarone. *Indian Heart J.* 69 707–713. 10.1016/j.ihj.2017.05.024 29174246 PMC5717288

[B206] Shi H. Kokoeva M. V. Inouye K. Tzameli I. Yin H. Flier J. S. (2006). TLR4 links innate immunity and fatty acid-induced insulin resistance. *J. Clin. Invest.* 116 3015–3025. 10.1172/JCI28898 17053832 PMC1616196

[B207] Stachon P. Heidenreich A. Merz J. Hilgendorf I. Wolf D. Willecke F. (2017). P2X7 deficiency blocks lesional inflammasome activity and ameliorates atherosclerosis in mice. *Circulation* 135 2524–2533. 10.1161/CIRCULATIONAHA.117.027400 28377486

[B208] Stein A. Wessling G. Deisenhofer I. Busch G. Steppich B. Estner H. (2008). Systemic inflammatory changes after pulmonary vein radiofrequency ablation do not alter stem cell mobilization. *Europace* 10 444–449. 10.1093/europace/eun041 18339614

[B209] Streitner F. Kuschyk J. Veltmann C. Brueckmann M. Streitner I. Brade J. (2007). Prospective study of interleukin-6 and the risk of malignant ventricular tachyarrhythmia in ICD-recipients–a pilot study. *Cytokine* 40 30–34. 10.1016/j.cyto.2007.07.187 17851087

[B210] Suganami T. Nishida J. Ogawa Y. (2005). A paracrine loop between adipocytes and macrophages aggravates inflammatory changes: role of free fatty acids and tumor necrosis factor alpha. *Arterioscler. Thromb. Vasc. Biol.* 25 2062–2068. 10.1161/01.ATV.0000183883.72263.13 16123319

[B211] Suganami T. Tanimoto-Koyama K. Nishida J. Itoh M. Yuan X. Mizuarai S. (2007). Role of the Toll-like receptor 4/NF-kappaB pathway in saturated fatty acid-induced inflammatory changes in the interaction between adipocytes and macrophages. *Arterioscler. Thromb. Vasc. Biol.* 27 84–91. 10.1161/01.ATV.0000251608.09329.9a 17082484

[B212] Sugishita K. Kinugawa K. Shimizu T. Harada K. Matsui H. Takahashi T. (1999). Cellular basis for the acute inhibitory effects of IL-6 and TNF- alpha on excitation-contraction coupling. *J. Mol. Cell Cardiol.* 31 1457–1467. 10.1006/jmcc.1999.0989 10423344

[B213] Sun X. L. Yuan J. F. Jin T. Cheng X. Q. Wang Q. Guo J. (2017). Physical and functional interaction of Snapin with Cav1.3 calcium channel impacts channel protein trafficking in atrial myocytes. *Cell Signal* 30 118–129. 10.1016/j.cellsig.2016.11.019 27915047

[B214] Szabo-Fresnais N. Lefebvre F. Germain A. Fischmeister R. Pomerance M. (2010). A new regulation of IL-6 production in adult cardiomyocytes by beta-adrenergic and IL-1 beta receptors and induction of cellular hypertrophy by Il-6 trans-signalling. *Cell. Signal.* 22 1143–1152. 10.1016/j.cellsig.2010.03.009 20227492

[B215] Szczepaniak L. S. Victor R. G. Orci L. Unger R. H. (2007). Forgotten but not gone: the rediscovery of fatty heart, the most common unrecognized disease in America. *Circ. Res.* 101 759–767. 10.1161/CIRCRESAHA.107.160457 17932333

[B216] Taga T. Kishimoto T. (1997). Gp130 and the interleukin-6 family of cytokines. *Annu. Rev. Immunol.* 15 797–819. 10.1146/annurev.immunol.15.1.797 9143707

[B217] Takeda K. Kaisho T. Akira S. (2003). Toll-like receptors. *Annu. Rev. Immunol.* 21 335–376. 10.1146/annurev.immunol.21.120601.141126 12524386

[B218] Tanaka T. Kanda T. Takahashi T. Saegusa S. Moriya J. Kurabayashi M. (2004). Interleukin-6-induced reciprocal expression of SERCA and natriuretic peptides mRNA in cultured rat ventricular myocytes. *J. Int. Med. Res.* 32 57–61. 10.1177/147323000403200109 14997707

[B219] Tanaka T. Narazaki M. Kishimoto T. (2017). Interleukin (IL-6) Immunotherapy. *Cold Spring Harb. Perspect. Biol.* 10:a028456. 10.1101/cshperspect.a028456 28778870 PMC6071487

[B220] Tian M. Dong M. Q. Chiu S. W. Lau C. P. Li G. R. (2006). Effects of the antifungal antibiotic clotrimazole on human cardiac repolarization potassium currents. *Br. J. Pharmacol.* 147 289–297. 10.1038/sj.bjp.0706590 16341233 PMC1751304

[B221] Timmers L. Sluijter J. P. Van Keulen J. K. Hoefer I. E. Nederhoff M. G. Goumans M. J. (2008). Toll-like receptor 4 mediates maladaptive left ventricular remodeling and impairs cardiac function after myocardial infarction. *Circ. Res.* 102 257–264. 10.1161/CIRCRESAHA.107.158220 18007026

[B222] Tsushima K. Bugger H. Wende A. R. Soto J. Jenson G. A. Tor A. R. (2018). Mitochondrial reactive oxygen species in lipotoxic hearts induce post-translational modifications of AKAP121, DRP1, and OPA1 that promote mitochondrial fission. *Circ. Res.* 122 58–73. 10.1161/CIRCRESAHA.117.311307 29092894 PMC5756120

[B223] Ucar H. I. Tok M. Atalar E. Dogan O. F. Oc M. Farsak B. (2007). Predictive significance of plasma levels of interleukin-6 and high-sensitivity C-reactive protein in atrial fibrillation after coronary artery bypass surgery. *Heart Surg. Forum* 10 E131–E135. 10.1532/HSF98.20061175 17597037

[B224] Ukena C. Mahfoud F. Kindermann I. Kandolf R. Kindermann M. Bohm M. (2011). Prognostic electrocardiographic parameters in patients with suspected myocarditis. *Eur. J. Heart Fail.* 13 398–405. 10.1093/eurjhf/hfq229 21239404

[B225] Vaez H. Rameshrad M. Najafi M. Barar J. Barzegari A. Garjani A. (2016). Cardioprotective effect of metformin in lipopolysaccharide-induced sepsis via suppression of toll-like receptor 4 (TLR4) in heart. *Eur. J. Pharmacol.* 772 115–123. 10.1016/j.ejphar.2015.12.030 26708162

[B226] Valli H. Ahmad S. Fraser J. A. Jeevaratnam K. Huang C. L. (2017). Pro-arrhythmic atrial phenotypes in incrementally paced murine Pgc1beta(-/-) hearts: effects of age. *Exp. Physiol.* 102 1619–1634. 10.1113/EP086589 28960529 PMC5725712

[B227] van der Lee K. A. Vork M. M. De Vries J. E. Willemsen P. H. Glatz J. F. Reneman R. S. (2000). Long-chain fatty acid-induced changes in gene expression in neonatal cardiac myocytes. *J. Lipid Res.* 41 41–47. 10627500

[B228] van Herpen N. A. Schrauwen-Hinderling V. B. (2008). Lipid accumulation in non-adipose tissue and lipotoxicity. *Physiol. Behav.* 94 231–241. 10.1016/j.physbeh.2007.11.049 18222498

[B229] van Niekerk G. Engelbrecht A. M. (2018). Inflammation-induced metabolic derangements or adaptation: an immunometabolic perspective. *Cytokine Growth Factor Rev.* 43 47–53. 10.1016/j.cytogfr.2018.06.003 29970338

[B230] Varro A. Nanasi P. P. Lathrop D. A. (1993). Potassium currents in isolated human atrial and ventricular cardiocytes. *Acta Physiol. Scand.* 149 133–142. 10.1111/j.1748-1716.1993.tb09605.x 8266802

[B231] Vicente R. Escalada A. Coma M. Fuster G. Sanchez-Tillo E. Lopez-Iglesias C. (2003). Differential voltage-dependent K+ channel responses during proliferation and activation in macrophages. *J. Biol. Chem.* 278 46307–46320. 10.1074/jbc.M304388200 12923194

[B232] Vilahur G. Badimon L. (2014). Ischemia/reperfusion activates myocardial innate immune response: the key role of the toll-like receptor. *Front. Physiol.* 5:496. 10.3389/fphys.2014.00496 25566092 PMC4270170

[B233] Villalonga N. David M. Bielanska J. Vicente R. Comes N. Valenzuela C. (2010). Immunomodulation of voltage-dependent K+ channels in macrophages: molecular and biophysical consequences. *J. Gen. Physiol.* 135 135–147. 10.1085/jgp.200910334 20100893 PMC2812499

[B234] Villegas S. Villarreal F. J. Dillmann W. H. (2000). Leukemia Inhibitory Factor and Interleukin-6 downregulate sarcoplasmic reticulum Ca2+ ATPase (SERCA2) in cardiac myocytes. *Basic Res. Cardiol.* 95 47–54. 10.1007/s003950050007 10752545

[B235] Wang J. Wang H. Zhang Y. Gao H. Nattel S. Wang Z. (2004). Impairment of HERG K(+) channel function by tumor necrosis factor-alpha: role of reactive oxygen species as a mediator. *J. Biol. Chem.* 279 13289–13292. 10.1074/jbc.C400025200 14973143

[B236] Wang Y. Qian Y. Fang Q. Zhong P. Li W. Wang L. (2017). Saturated palmitic acid induces myocardial inflammatory injuries through direct binding to TLR4 accessory protein MD2. *Nat. Commun.* 8:13997. 10.1038/ncomms13997 28045026 PMC5216130

[B237] Wang Y. Qian Y. Fang Q. Zhong P. Li W. Wang L. (2018). Author Correction: saturated palmitic acid induces myocardial inflammatory injuries through direct binding to TLR4 accessory protein MD2. *Nat. Commun.* 9:16185. 10.1038/ncomms16185 29553572 PMC5859348

[B238] Wang Y. Zankov D. P. Jiang M. Zhang M. Henderson S. C. Tseng G. N. (2013). [Ca2+]i elevation and oxidative stress induce KCNQ1 protein translocation from the cytosol to the cell surface and increase slow delayed rectifier (IKs) in cardiac myocytes. *J. Biol. Chem.* 288 35358–35371. 10.1074/jbc.M113.504746 24142691 PMC3853284

[B239] Watanabe Y. Nagai Y. Takatsu K. (2013). Activation and regulation of the pattern recognition receptors in obesity-induced adipose tissue inflammation and insulin resistance. *Nutrients* 5 3757–3778. 10.3390/nu5093757 24064574 PMC3798933

[B240] Wehrens X. H. Rossenbacker T. Jongbloed R. J. Gewillig M. Heidbuchel H. Doevendans P. A. (2003). A novel mutation L619F in the cardiac Na+ channel SCN5A associated with long-QT syndrome (LQT3): a role for the I-II linker in inactivation gating. *Hum. Mutat.* 21:552. 10.1002/humu.9136 12673799

[B241] Wen H. Gris D. Lei Y. Jha S. Zhang L. Huang M. T. (2011). Fatty acid-induced NLRP3-ASC inflammasome activation interferes with insulin signaling. *Nat. Immunol.* 12 408–415. 10.1038/ni.2022 21478880 PMC4090391

[B242] Willebrords J. Crespo Yanguas S. Maes M. Decrock E. Wang N. Leybaert L. (2016). Connexins and their channels in inflammation. *Crit. Rev. Biochem. Mol. Biol* 51 413–439. 10.1080/10409238.2016.1204980 27387655 PMC5584657

[B243] Wit A. L. (2018). Afterdepolarizations and triggered activity as a mechanism for clinical arrhythmias. *Pacing Clin. Electrophysiol.* 10.1111/pace.13419 [Epub ahead of print]. 29920724

[B244] Wong F. S. Wen L. (2008). Toll-like receptors and diabetes. *Ann. N. Y. Acad. Sci.* 1150 123–132. 10.1196/annals.1447.063 19120280

[B245] Wong S. C. Fukuchi M. Melnyk P. Rodger I. Giaid A. (1998). Induction of cyclooxygenase-2 and activation of nuclear factor-kappaB in myocardium of patients with congestive heart failure. *Circulation* 98 100–103. 10.1161/01.CIR.98.2.100 9679714

[B246] Wu N. Xu B. Xiang Y. Wu L. Zhang Y. Ma X. (2013). Association of inflammatory factors with occurrence and recurrence of atrial fibrillation: a meta-analysis. *Int. J. Cardiol.* 169 62–72. 10.1016/j.ijcard.2013.08.078 24095158

[B247] Xiao Y. F. Ke Q. Wang S. Y. Yang Y. Chen Y. Wang G. K. (2004). Electrophysiologic properties of lidocaine, cocaine, and n-3 fatty-acids block of cardiac Na+ channels. *Eur. J. Pharmacol.* 485 31–41. 10.1016/j.ejphar.2003.11.042 14757121

[B248] Yagyu H. Chen G. Yokoyama M. Hirata K. Augustus A. Kako Y. (2003). Lipoprotein lipase (LpL) on the surface of cardiomyocytes increases lipid uptake and produces a cardiomyopathy. *J. Clin. Invest.* 111 419–426. 10.1172/JCI16751 12569168 PMC151861

[B249] Yamasaki K. Taga T. Hirata Y. Yawata H. Kawanishi Y. Seed B. (1988). Cloning and expression of the human interleukin-6 (BSF-2/IFN beta 2) receptor. *Science* 241 825–828. 10.1126/science.3136546 3136546

[B250] Yang S. Zheng R. Hu S. Ma Y. Choudhry M. A. Messina J. L. (2004). Mechanism of cardiac depression after trauma-hemorrhage: increased cardiomyocyte IL-6 and effect of sex steroids on IL-6 regulation and cardiac function. *Am. J. Physiol. Heart Circ. Physiol.* 287 H2183–H2191. 10.1152/ajpheart.00624.2003 15475534

[B251] Yin J. Wang Y. Hu H. Li X. Xue M. Cheng W. (2017). P2X7 receptor inhibition attenuated sympathetic nerve sprouting after myocardial infarction via the NLRP3/IL-1beta pathway. *J. Cell Mol. Med.* 21 2695–2710. 10.1111/jcmm.13185 28470940 PMC5661108

[B252] Youssef-Elabd E. M. Mcgee K. C. Tripathi G. Aldaghri N. Abdalla M. S. Sharada H. M. (2012). Acute and chronic saturated fatty acid treatment as a key instigator of the TLR-mediated inflammatory response in human adipose tissue, *in vitro*. *J. Nutr. Biochem.* 23 39–50. 10.1016/j.jnutbio.2010.11.003 21414768 PMC3243902

[B253] Yu X. W. Chen Q. Kennedy R. H. Liu S. J. (2005). Inhibition of sarcoplasmic reticular function by chronic interleukin-6 exposure via iNOS in adult ventricular myocytes. *J. Physiol.* 566 327–340. 10.1113/jphysiol.2005.086686 15845578 PMC1464756

[B254] Zhang F. W. Tong J. Yan Y. S. Chen Q. Q. Zhao X. P. (2018). omega-3 polyunsaturated fatty acid postconditioning protects the isolated perfused rat heart from ischemia-reperfusion injury. *Cardiorenal Med.* 8 173–182. 10.1159/000487490 29642067 PMC6167714

[B255] Zhang Z. He Y. Tuteja D. Xu D. Timofeyev V. Zhang Q. (2005). Functional roles of Cav1.3*(alpha*1D) calcium channels in atria: insights gained from gene-targeted null mutant mice. *Circulation* 112 1936–1944. 10.1161/CIRCULATIONAHA.105.540070 16172271

[B256] Zhao L. Cheng G. Jin R. Afzal M. R. Samanta A. Xuan Y. T. (2016a). Deletion of interleukin-6 attenuates pressure overload-induced left ventricular hypertrophy and dysfunction. *Circ. Res.* 118 1918–1929. 10.1161/CIRCRESAHA.116.308688 27126808 PMC4902783

[B257] Zhao Y. Sun Q. Zeng Z. Li Q. Zhou S. Zhou M. (2016b). Regulation of SCN3B/scn3b by Interleukin 2 (IL-2): IL-2 modulates SCN3B/scn3b transcript expression and increases sodium current in myocardial cells. *BMC Cardiovasc. Disord.* 16:1. 10.1186/s12872-015-0179-x 26728597 PMC4700781

[B258] Zhou B. Rao L. Peng Y. Wang Y. Li Y. Gao L. (2009). Functional polymorphism of the NFKB1 gene promoter is related to the risk of dilated cardiomyopathy. *BMC Med. Genet.* 10:47. 10.1186/1471-2350-10-47 19480714 PMC2692851

[B259] Zhou W. Cayabyab F. S. Pennefather P. S. Schlichter L. C. Decoursey T. E. (1998). HERG-like K+ channels in microglia. *J. Gen. Physiol.* 111 781–794. 10.1085/jgp.111.6.781 9607936 PMC2217149

